# Computational Mapping of Hedgehog Pathway Kinase Module Predicts Node-Specific Craniofacial Phenotypes

**DOI:** 10.3390/genes17040433

**Published:** 2026-04-08

**Authors:** Kosi Gramatikoff, Miroslav Stoykov, Karl Hörmann, Mario Milkov

**Affiliations:** 1 Research Institute, Medical University “Prof. Dr. Paraskev Stoyanov”–Varna, 55 Marin Drinov Str., 9002 Varna, Bulgaria; 2 Department of Dental Material Science and Prosthetic Dental Medicine, Faculty of Dental Medicine, Medical University “Prof. Dr. Paraskev Stoyanov”–Varna, 84 Tsar Osvoboditel Blvd, 9002 Varna, Bulgaria; miroslav.stoikov93@gmail.com; 3 Mannheim University Hospital, Fichtestraße 2, D-68165 Mannheim, Germany; karl@hoermann-hno.de

**Keywords:** craniofacial malformations, Sonic Hedgehog, molecular docking, network medicine, neural crest, developmental toxicology, CK1δ, PINK1, morphogenic modules

## Abstract

Background/Objectives: Craniofacial malformations such as orofacial clefts affect ~1 in 700 births; 40–60% lack clear genetic etiology, and many exhibit asymmetry and variable expressivity unexplained by classical Sonic Hedgehog (SHH) morphogen gradient models. We investigated whether integrated molecular modules linking morphogen signaling with metabolic stress responses may better account for craniofacial developmental outcomes. Methods: Sequential UniProt gene set integration identified 186 candidate craniofacial regulators. STRING network analysis revealed modular architecture. Molecular docking profiled 17 compounds against SMO, CK1δ, PINK1, and TIE2 (control). Pathway reconstruction integrated the SHH–CK1δ–HIF1A–HEY1–PINK1 axis with in-silico-predicted CK1δ phosphorylation sites on SMO (S615, T593, S751), HIF1A (Ser247), and GLI1/2/3 transcription factors. A developmental decision tree mapped affinity profiles to node-specific phenotype hypotheses. Results: CK1δ and PINK1 emerged as candidate nodes coupling morphogen signaling with mitochondrial quality control. Cross-docking showed preferential binding to developmental kinases (CK1δ: −8.34 kcal/mol; PINK1: −8.80 kcal/mol) versus TIE2 control (−6.76 kcal/mol; *p* < 0.001). Pathway reconstruction suggested that CK1δ-mediated Ser247 phosphorylation of HIF1A disrupts ARNT dimerization, redirecting HIF1A toward ARNT-independent HEY1 induction and consequent PINK1 suppression. Based on computed profiles, node-specific associations were proposed as computational hypotheses: SMO perturbation → midline defects; CK1δ → facial asymmetry/clefting; PINK1 → mandibular hypoplasia. Multi-target compounds (e.g., purmorphamine, taladegib) generated composite phenotype predictions consistent with clinical complexity. Conclusions: This strictly in silico study identifies candidate integrated morphogenic modules whose multi-node perturbation may underlie anatomically specific craniofacial malformation patterns. Node–phenotype associations are prioritized computational hypotheses requiring experimental validation; if confirmed, the framework could inform developmental toxicity assessment, therapeutic design, and reclassification of idiopathic craniofacial anomalies.

## 1. Introduction

Craniofacial development represents one of the most complex patterning processes in vertebrate embryogenesis, requiring precise integration of morphogen gradients, cellular metabolism, and environmental cues to establish the intricate three-dimensional architecture of the face and skull [1]. Congenital craniofacial anomalies affect approximately 1 in 700 live births, representing a substantial clinical and public health burden [2]. Despite decades of genetic and developmental biology research, the molecular etiology remains unclear in 40–60% of cases [3], highlighting critical gaps in our understanding of craniofacial morphogenesis and its vulnerability to perturbation.

Among the signaling pathways orchestrating craniofacial patterning, Sonic Hedgehog (SHH) plays a central role in regulating midline specification, neural crest cell survival, and facial prominence development [4,5]. Classical models emphasize SHH function as a morphogen, whereby graded Smoothened (SMO) receptor activity is transduced through GLI transcription factors to establish spatially ordered gene expression programs [6]. Disruption of this canonical axis typically yields holoprosencephaly spectrum disorders or uniform truncation of craniofacial structures [7], phenotypes well-explained by loss of morphogen gradient formation.

However, accumulating clinical observations challenge a purely morphogen-centric view of SHH signaling. Many craniofacial malformations display marked asymmetry, regional selectivity, and variable expressivity, often in the absence of identifiable pathogenic variants in canonical SHH pathway genes [8,9]. Phenotypes such as isolated cleft lip/palate, Pierre Robin sequence, craniofacial microsomia, and mandibular hypoplasia show strong modulation by maternal metabolic state, hypoxic exposure, and environmental stressors [10,11], suggesting that developmental outcomes depend on factors beyond simple genetic loss of morphogen signaling. These observations indicate that SHH pathway function may be context-dependent, with failure modes distinct from classical morphogen gradient collapse.

**Figure 1:**
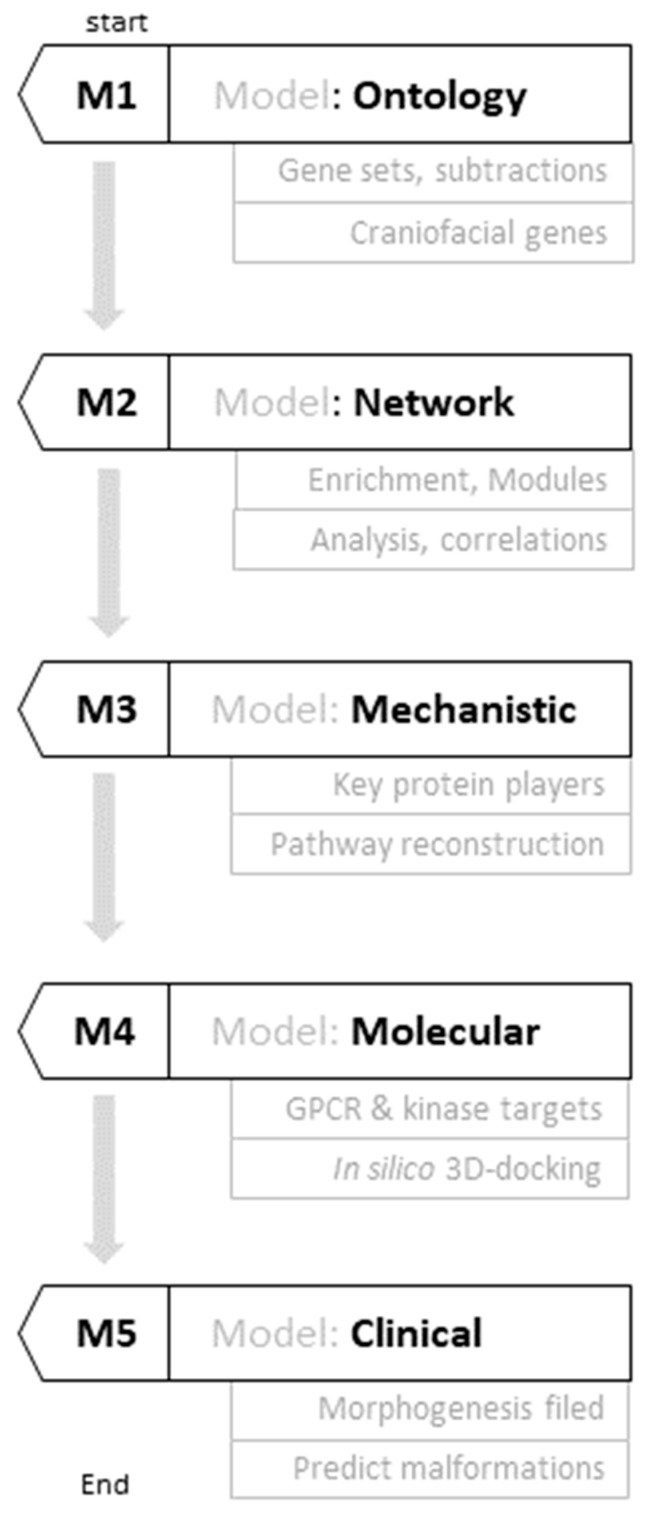
*Five-tier computational workflow for predicting neural crest loss and craniofacial malformations through Sonic Hedgehog kinase module analysis*. The workflow integrates five progressively refined modeling approaches: (M1) Ontology-based systematic curation of craniofacial development gene sets from UniProt and KEGG databases; (M2) Network enrichment analysis revealing convergent SHH and WNT signaling axes with GPCR and kinase hubs; (M3) Mechanistic pathway reconstruction mapping signal transduction cascades governing neural crest cell fate; (M4) Molecular characterization through in silico 3D docking of ligand–protein-binding affinities; and (M5) Clinical integration establishing computational biomarkers for craniofacial anomalies. This hierarchical framework enables systematic progression from genomic data through molecular mechanisms to phenotype prediction.

Challenge 1: Establishing Unified Craniofacial Gene Ontology. A fundamental obstacle to understanding craniofacial development has been the absence of a unified, comprehensive gene ontology that captures the full molecular landscape governing facial morphogenesis. Existing pathway databases focus on canonical signaling cascades (WNT, BMP, FGF, SHH) but fail to integrate stress-responsive kinases, metabolic regulators, and context-dependent modifiers that influence developmental outcomes. The authors addressed this challenge through a two-stage sequential integration strategy using the UniProt Knowledgebase [12], the most extensively curated protein annotation resource. Initial intersection of neural crest genes (n = 167), embryonic genes (n = 641), and craniofacial-specific genes (n = 19) identified SHH and WNT pathways as critical primers. Refined integration with the complete human kinome (n = 474) and developmental genes (n = 5494) converged on 186 high-confidence candidates, uniquely identifying casein kinase 1 delta (CK1δ) at the intersection of developmental processes and kinase signaling (Figure 1, M1). This systematic, ontology-driven approach indicated that craniofacial morphogenesis may depend not only on morphogen pathways but also on kinases integrating circadian regulation, metabolic timing, and stress responses.

Network-based analysis of the 186-gene set using the STRING database [13] revealed modular organization with SHH and HIF1A anchoring a core regulatory hub that interfaces with CK1δ, PTEN-induced kinase 1 (PINK1), and GLI transcription factors. PINK1, a mitochondrial serine/threonine kinase essential for mitophagy and quality control [14], emerged as an unexpected candidate craniofacial development regulator. While PINK1 is best known for its role in Parkinson’s disease pathogenesis [15], its function in neural crest cells—which undergo dramatic metabolic reprogramming during migration and are exquisitely sensitive to oxidative stress [16]—provides mechanistic rationale for its developmental annotation. CK1δ, a multi-functional kinase implicated in circadian regulation and WNT signaling [17], has been shown to phosphorylate SMO to promote pathway activation [18], while also modulating HIF1A activity and mitochondrial dynamics, positioning it at the intersection of morphogen signaling and cellular stress biology.

Challenge 2: Applying Network Medicine to Model Craniofacial Pathologies. Traditional developmental biology approaches focus on single-gene perturbations and linear pathway models, which are poorly suited for understanding complex, context-dependent phenotypes arising from multi-node network perturbations. The authors applied network medicine principles [19] to model craniofacial pathologies through systematic molecular docking analysis of 17 compounds against four protein targets: SMO, CK1δ, PINK1, and TIE2/TEK (non-developmental control kinase). This cross-docking approach [20], conventionally employed for drug repurposing, was repurposed here to map integrated module architecture and explore how perturbations may propagate through the SHH–CK1δ–PINK1 network to produce anatomically specific malformations.

The systematic binding affinity analysis revealed that SMO-targeting compounds engage CK1δ and PINK1 with comparable or stronger affinity than their intended target. Mean docking scores were: −8.34 kcal/mol (CK1δ), −8.80 kcal/mol (PINK1), −7.56 kcal/mol (SMO), and −6.76 kcal/mol (TIE2), with highly significant preferential binding to developmental kinases versus the control (*p* < 0.001 for all comparisons). This polypharmacology was not compound-specific but extended across both SMO-directed agents and downstream modulators, suggesting genuine affinity for kinases co-expressed during embryogenesis rather than non-specific promiscuity. Compounds with strongest dual CK1δ/PINK1 engagement (purmorphamine, taladegib, vismodegib) are precisely those associated with craniofacial developmental toxicity in clinical and preclinical studies [21,22], an observation that supports, though does not by itself establish, the biological relevance of these computational findings.

Pathway reconstruction identified a candidate stress-responsive module in which CK1δ phosphorylates both SMO, at in-silico-predicted sites S615, T593, S751, requiring experimental confirmation, and HIF1A at Ser247 within the PAS-B domain [23]. The functional consequence of Ser247 phosphorylation is mechanistically distinct from simple HIF1A stabilization: phosphorylation at this site impairs HIF1A dimerization with its canonical partner aryl hydrocarbon receptor nuclear translocator (ARNT), thereby specifically suppressing ARNT-dependent, hypoxia response element (HRE)-driven transcription [23]. Critically, this ARNT-disrupting phosphorylation does not simply inactivate HIF1A but redirects its transcriptional activity: phospho-Ser247 HIF1A retains the capacity to engage non-canonical transcriptional partners and to regulate target genes independently of the HRE/ARNT axis [23]. In this context, published experimental evidence demonstrates that HIF1A directly induces HEY1, a Notch-pathway transcriptional repressor, through a mechanism that is not dependent on classical ARNT-mediated HRE binding [23,24]. HEY1 in turn suppresses PINK1 expression [24], limiting mitochondrial quality control capacity precisely when proliferating neural crest cells require robust mitophagy. The Ser247 motif identified in HIF1A is also present in other PAS-B domain-containing proteins, including HIF2A, HIF3A, NPAS2/CLOCK, and AXIN2, raising the possibility that CK1δ-mediated phosphorylation at this motif may coordinately redirect transcriptional activity across multiple members of the HIF and circadian regulator families, a hypothesis that requires experimental investigation.

The coupling of these nodes creates a proposed pathological architecture in which perturbation of any one node may propagate through feedback interactions to compromise both morphogen signaling fidelity and cellular stress resilience. Under normoxic conditions, partial perturbations are predicted to be buffered; under hypoxic conditions, a defining feature of embryonic craniofacial primordia [25], SHH signaling may shift from a morphogenetic to survival-supporting function, with multi-node inhibition potentially producing selective neural crest apoptosis rather than uniform tissue loss. These are computational inferences from the network model and await experimental validation.

To translate binding affinity profiles into anatomically organized phenotype hypotheses, a developmental decision tree was constructed mapping compounds to craniofacial malformation patterns based on node-specific perturbation. SMO-dominant compounds are hypothesized to affect midline patterning (holoprosencephaly, frontal bossing, median clefts); CK1δ-dominant compounds are hypothesized to disrupt bilateral symmetry (facial asymmetry, craniosynostosis, maxillofacial clefts); and PINK1-dominant compounds are hypothesized to impair mandibular development (micrognathia, retrognathia, Treacher Collins-like features). Compounds appearing on multiple branches (purmorphamine: all three nodes; cyclopamine, taladegib: two nodes) are predicted to produce composite phenotypes, while node-selective compounds are predicted to generate more anatomically restricted malformations. These node–phenotype associations are presented as prioritized computational hypotheses requiring experimental confirmation, not as established conclusions; they are offered here to frame testable predictions for future in vitro and in vivo studies.

Downstream of SMO, the GLI transcription factors (GLI1, GLI2, GLI3) are hypothesized to be subject to CK1-family phosphorylation at sites identified by in silico cross-tool consensus analysis (see Materials and Methods). Candidate sites identified for the present study are: GLI1: S640; GLI2: S792, S808, S820, S1014; GLI3: S78, S445, S880, S910. As discussed in the Materials and Methods, these sites were predicted using tools queried primarily for CK1α; their assignment to CK1δ is based on shared substrate motif logic (pSer/pThr–X–X–Ser/Thr) and represents a computational inference rather than direct experimental evidence of isoform-specific phosphorylation.

Study Objectives and Significance. The study demonstrates that: (1) systematic gene ontology integration reveals kinase-mediated coupling between morphogen signaling and metabolic stress responses in craniofacial development; (2) network-based approaches identify candidate integrated modules (SHH–CK1δ–PINK1) governing context-dependent developmental outcomes; (3) cross-docking computational analysis, when applied to map multi-node architecture rather than identify drug candidates, generates prioritized, testable hypotheses about craniofacial malformation patterns based on compound binding affinity profiles; and (4) the resulting framework offers a mechanistic basis for understanding asymmetric, variable, and environmentally sensitive phenotypes that dominate clinical practice but remain poorly explained by gene-centric approaches. All findings are strictly in silico; the translational applications discussed in this study, including mechanism-based developmental toxicology, rational design of safer therapeutic agents, and potential etiologic reclassification of idiopathic craniofacial anomalies, are presented as long-term possibilities contingent on experimental validation of the core computational model.

## 2. Materials and Methods

### 2.1. Data Sources and Gene Annotations

#### Protein and Gene Databases

All protein-coding gene entries used in our gene set collections were obtained from the UniProt Knowledgebase (UniProtKB) [26], the world’s leading high-quality, freely accessible database of protein sequence and functional information. UniProtKB curates experimentally verified protein sequences and functional annotations and maintains non-redundant reference proteomes, providing comprehensive, well-standardized, and up-to-date protein/gene data. The curated nature of UniProt, combined with automated annotation pipelines for unreviewed entries and continued expert/manual curation of literature data, ensures both breadth and depth of coverage for human and disease-relevant proteins, making it uniquely suited for large-scale network data integration studies.

### 2.2. Gene Set Assembly and Integration

#### 2.2.1. Initial Gene Set Assembly (Stage V1)

The authors conducted a two-stage sequential gene set integration analysis to identify key regulators of craniofacial development. In the first stage (V1), the authors assembled three primary gene sets: (1) neural-crest-associated genes (n = 167), validated through concordance between Gene Ontology Biological Process (GO-BP) and WikiPathways databases [27]; (2) embryonic development genes (n = 641); and (3) craniofacial-specific genes (n = 19). Initial candidates HIF1A and SMO were identified within the neural crest gene set, while SHH was identified within the craniofacial subset.

#### 2.2.2. Pathway Enrichment and Primer Identification

Functional enrichment analysis of the V1 gene sets was performed using the Kyoto Encyclopedia of Genes and Genomes (KEGG) pathway database [27]. The analysis identified WNT and SHH signaling pathways as significantly enriched and established these as pathway primers for subsequent refinement. Complete pathway annotations for WNT and SHH signaling were extracted from KEGG and mapped to the human kinome to identify druggable targets [27].

#### 2.2.3. Refined Gene Set Integration (Stage V2)

In the second stage (V2), the authors integrated four complementary gene sets: (1) general developmental genes (n = 5494); (2) facial-development-specific genes (n = 205); (3) the complete human kinome (n = 474); and (4) the comprehensive WNT and SHH pathway gene sets identified in V1. Venn diagram analysis revealed substantial overlap between developmental processes and kinase signaling, with 255 genes (54% of the kinome) shared between developmental and kinome datasets. Intersection with the WNT pathway identified 14 overlapping genes, while intersection with the SHH pathway revealed 10 overlapping genes. The critical intersection of all analyzed sets uniquely identified casein kinase 1 delta (CK1δ; also detected with three CK1γ isoforms) as a central regulator. Additional key kinases tyrosine kinase with immunoglobulin and epidermal growth factor homology domains 2 (TIE2, TEK) and PTEN-induced kinase 1 (PINK1) were identified through this integration strategy.

#### 2.2.4. Final Candidate Gene Set

The sequential filtering and integration strategy resulted in the identification of 186 unique genes with convergent evidence for involvement in craniofacial development. This refined gene set represents the intersection of developmental biology, kinase signaling networks, and established morphogenetic pathways (WNT and SHH), providing a high-confidence list of candidate regulators and potential therapeutic targets for craniofacial disorders. Venn diagrams were generated to visualize gene set overlaps and intersections at each analytical stage, with percentage calculations for kinome representation performed relative to the total human kinome (474 genes).

### 2.3. Protein–Protein Interaction Network Analysis

#### 2.3.1. Network Construction

The 186 candidate genes identified from the sequential gene set integration analysis were submitted to the Search Tool for the Retrieval of Interacting Genes/Proteins (STRING) database (https://string-db.org/) to construct a comprehensive protein–protein interaction (PPI) network. STRING systematically integrates known and predicted associations—both direct physical interactions and indirect functional associations (e.g., co-expression, shared pathway membership, genomic context, literature co-mentions)—by collating evidence from experimental databases, curated pathway resources, computational predictions, and text mining. For each protein pair, STRING assigns a confidence score reflecting the aggregated weight of evidence across multiple channels. Network construction parameters included a minimum required interaction score of 0.4 (medium confidence), with all active interaction sources enabled. Only associations above the confidence threshold were retained to define network edges. The resulting network was visualized with evidence-type-specific edge coloring: purple edges represent experimentally determined interactions, green edges indicate gene co-expression patterns, pink edges denote other functional associations.

#### 2.3.2. Module Identification and Characterization

Functional modules within the master network were identified using the Markov Cluster Algorithm (MCL) clustering method implemented in STRING with an inflation parameter of 3.0. Six major modules were detected and designated Module 1 through Module 6 based on their spatial organization and functional coherence. Module boundaries were determined by network topology, with modules defined as densely connected subgraphs with sparse connections between modules. Module 1 was identified as the central hub based on its position in the network layout and high betweenness centrality of constituent nodes. This enrichment identifies ontology categories and functional modules overrepresented in the network compared to genome-wide background expectations, enabling assignment of module labels and boundaries based on ontology signatures.

#### 2.3.3. Hybrid Module Extraction

A hybrid module was extracted by selecting genes from both Module 1 and Module 2 that exhibited direct interactions or shared functional annotations related to SHH pathway signaling. Key anchor genes for module extraction included SHH, PTCH1, SMO, GLI1, GLI2, GLI3, HIF1A, CSNK1D (CK1δ), and PINK1. The extracted subnetwork was reanalyzed to identify unexpected connections and pathway cross-talk. Node coloring in the hybrid module visualization reflects ontological category membership, with multi-colored nodes representing genes annotated across multiple functional categories as determined by enrichment analysis.

#### 2.3.4. Functional Enrichment Analysis

Functional enrichment analysis of the hybrid module was performed using STRING enrichment tools, interrogating five complementary ontology databases: (1) Gene Ontology Molecular Function (MF-GO), (2) Gene Ontology Biological Process (BP-GO), (3) KEGG Pathways, (4) WikiPathways, and (5) Human Phenotype Ontology (Monarch Initiative). For each ontology, the authors calculated enrichment statistics including observed gene count, background gene count, network strength (ratio of observed to expected connections), signal strength (observed/expected ratio), and false discovery rate (FDR)-corrected *p*-values using the Benjamini–Hochberg method. Statistical significance was defined as FDR < 0.05. Color assignments for each ontology category (purple for MF-GO DNA binding, green for transcription regulator activity, pink for KEGG mitophagy, yellow for Wiki hedgehog pathway, and red for Human Phenotype cleft lip) were applied to corresponding nodes in the hybrid module visualization, with nodes participating in multiple enriched categories displayed as multi-colored.

#### 2.3.5. Disease Association Analysis

Disease phenotypes associated with the 186-gene network were retrieved from the Online Mendelian Inheritance in Man (OMIM) database through STRING. Phenotype descriptions were extracted, processed to remove common words and standardized medical terminology, and compiled for frequency analysis. A word cloud was generated with word size proportional to frequency of occurrence across all disease descriptions. Words appearing fewer than 3 times were excluded from visualization to highlight the most prevalent phenotypic associations. All enrichment statistics were calculated using hypergeometric tests with FDR correction for multiple testing. Network topology statistics including node degree, betweenness centrality, and clustering coefficients were calculated using STRING built-in algorithms.

### 2.4. Mechanistic Pathway Reconstruction

#### 2.4.1. Pathway Architecture and Literature Curation—In Silico Prediction of CK1δ Phosphorylation Sites in Human Smoothened (SMO) and GLI Transcription Factors

##### Rationale for In Silico Phosphosite Prediction

Casein kinase 1δ (CK1δ; gene symbol CSNK1D, UniProt O15049) is one of the seven mammalian CK1 isoforms and shares high sequence and structural identity with casein kinase 1α (CK1α; CSNK1A1, UniProt P48729). Pairwise alignment of the two full-length human protein sequences using DIALIGN-TX and MAFFT (default parameters) yielded 75.0% amino acid identity over a 300-residue overlap. When alignment was restricted to the kinase/catalytic domain, identity increased to 82.8%. Comparative analysis of the available crystal structures (CK1δ: PDB 6GZD; CK1α: PDB 3UYT) confirmed that the catalytic cores are structurally superimposable (root-mean-square deviation < 0.5 Å over Cα atoms), whereas sequence divergence is confined to the N- and C-terminal regulatory regions. These observations are consistent with the accepted view that CK1α and CK1δ recognize essentially identical consensus phosphorylation motifs—principally primed Ser/Thr motifs of the form pSer/pThr–X–X–Ser/Thr—and can phosphorylate overlapping sets of substrates [17,28].

Because published experimental evidence places CK1α as the principal kinase responsible for SMO activation in Hedgehog (Hh) signaling [18,29], and because reviewers have queried whether CK1δ can phosphorylate human SMO at equivalent sites, we performed a systematic, multi-tool in silico prediction to identify candidate CK1δ phosphorylation sites within the human SMO protein. The objective was to determine whether residues previously associated with CK1-mediated SMO regulation are also predicted as CK1δ targets by independent computational approaches.

##### Query Protein Sequences

The canonical full-length amino acid sequence of human Smoothened (SMO; UniProt Q99835, isoform 1, 787 amino acids) was downloaded from the UniProt Knowledgebase (https://www.uniprot.org/uniprotkb/Q99835/entry, accessed on 5 April 2026) in FASTA format. For the downstream analysis of GLI transcription factors, the canonical sequences of human GLI1 (UniProt P08151), GLI2 (UniProt P10070), and GLI3 (UniProt P10071) were retrieved from UniProt in the same manner. All sequences were used as query inputs for phosphorylation prediction without modification.

##### Selection and Rationale for Phosphorylation Prediction Tools

Seven publicly accessible phosphoproteomics prediction tools were selected on the basis of complementary algorithmic foundations, peer-reviewed validation, and high visibility in the field (Table 1). Tool prominence was further confirmed by independent Google Scholar search ranking using the terms “phosphoprotein prediction” and “phosphoprotein prediction online tool”, under which Kinexus PhosphoNET and PhosphoSitePlus consistently ranked first and second/third, respectively. By combining tools that rely on kinase–substrate association scoring, neural network sequence analysis, pattern/motif recognition, and curated experimental databases, we aimed to identify phosphosites supported by convergent evidence across multiple independent methodologies.

##### Prediction Procedure

Each tool was queried with the human SMO FASTA sequence using its default settings. Where kinase-specific queries were supported (KP, NP, GP, PN), CK1δ (CSNK1D) was specified as the target kinase; for tools that do not distinguish CK1 isoforms (PP, PS, UP), all CK1 family entries were retrieved and manually filtered to include only those predictions consistent with classical CK1 primed or acidic-cluster consensus motifs. Serine (S) and threonine (T) residues were considered candidate phosphosites. Tyrosine predictions were excluded from the current analysis. Each tool was run independently, and the resulting lists of predicted phosphoresidues were compiled into a cross-tool comparison matrix. The same procedure was subsequently applied to GLI1, GLI2, and GLI3 sequences (see Section “Extension of In Silico Analysis to Downstream GLI Transcription Factors”).

##### Consensus Site Definition

A phosphosite was classified as a high-confidence consensus prediction if it was independently identified by three or more of the seven tools. This threshold was chosen to balance sensitivity (recovery of genuine CK1δ substrates) against specificity (exclusion of tool-specific artefacts). Sites identified by only one or two tools were recorded but not considered further in the primary analysis.

##### Results of Cross-Tool Consensus Analysis—SMO

Cross-referencing predictions across all seven tools identified three residues that surpassed the consensus threshold (Table 2): Thr593, which was predicted by all seven tools; Ser615, predicted by five of seven tools; and Ser751, predicted by three of seven tools. Together, T593, S615, and S751 constitute the high-confidence set of candidate CK1δ phosphorylation sites in human SMO identified by this in silico approach.

Note on Ser683:

Ser683 of human SMO has been cited in the literature as a CK1δ-dependent phosphorylation site acting downstream of PKC-mediated priming [29,30]. However, Ser683 did not emerge from the current cross-tool consensus analysis; its absence from the prediction set most likely reflects the priming-dependent nature of this event, which several tools do not model explicitly. The present analysis does not exclude Ser683 as a CK1δ target; rather, it underscores the distinction between computationally predicted de novo sites (T593, S615, S751) and experimentally inferred priming-dependent sites (S683).

##### Structural and Functional Context—SMO

The three consensus sites map to the intracellular cytoplasmic tail and loop regions of SMO (predicted based on the transmembrane topology reported in UniProt Q99835), consistent with accessibility to cytoplasmic kinases. T593 and S615 fall within a region of the intracellular C-terminal domain previously implicated in CK1-mediated regulation of SMO activity [18]. S751 is located in the distal C-terminal tail. The predicted CK1 recognition motifs surrounding each site were inspected manually for compatibility with the canonical CK1 consensus (pSer/pThr–X–X–Ser/Thr or acidic cluster–Ser/Thr), and all three residues are flanked by sequence contexts consistent with CK1δ substrate recognition.

##### Extension of In Silico Analysis to Downstream GLI Transcription Factors

The same seven-tool in silico pipeline described for SMO (Section 2.3, Section 2.4 and Section 2.5) was applied to the three human GLI transcription factors—GLI1, GLI2, and GLI3—that function as principal transcriptional effectors of the Hedgehog/SMO signaling axis. The objective was to determine whether CK1-family consensus motifs are present at specific residues of each GLI protein and whether the same motif logic that supports CK1δ phosphorylation of SMO extends to downstream pathway components.

Predicted phosphosites were accepted as candidate CK1 targets if they satisfied the canonical primed motif pSer/pThr–X–X–Ser/Thr, as defined by Prosite pattern matching and independently corroborated by at least one additional tool. Sites identified primarily by CK1α-specific queries were retained in the analysis where the flanking sequence context was judged equivalent to the CK1δ consensus, consistent with the shared substrate-recognition logic established in Section 2.1 and Section 2.3. Sites bearing non-canonical motifs (e.g., CK2α acidic-cluster patterns at GLI3 S78 and S445) were recorded separately, with CK1δ compatibility noted as a secondary prediction.

The cross-tool analysis identified a total of nine candidate phosphoresidues across the three GLI proteins (Table 3). In GLI1, a single high-confidence site was identified at Ser640, supported by multiple tools and conforming to the canonical CK1 primed motif. In GLI2, four sites were identified: S792 emerged as the highest-confidence prediction based on the top NetPhos 3.1 score and multi-tool support; S820 and S863 share an identical surrounding sequence context, as confirmed by Prosite pattern matching and Kinexus scoring, making them effectively equivalent candidate sites generated by local sequence repetition; S1014 was predicted by CK1α-directed queries and is considered a candidate CK1δ site on the basis of shared motif compatibility. S808 was captured by a subset of tools at subthreshold confidence and is reported in parentheses as a secondary prediction pending further validation. In GLI3, S880 and S910 were identified with high confidence; both residues are flanked by the Prosite-defined SseaS sequence pattern, which is also present at equivalent positions in GLI2 and in Period circadian regulator 3 (PER3), an established CK1δ substrate, lending additional credibility to the prediction. S78 and S445 in GLI3 responded to ischemia-associated queries and were initially flagged as CK1α/CK2α sites; their sequence contexts show partial, non-canonical compatibility with CK1δ consensus motifs and are therefore listed as secondary candidate sites.

The identification of CK1-consensus phosphosites in all three GLI transcription factors is consistent with the established role of CK1 family members in regulating GLI/Ci activator function [30,31] and supports a model in which CK1δ, acting through the same substrate recognition logic that governs SMO phosphorylation, participates in the posttranslational control of Hedgehog pathway output at the level of the transcriptional effectors. The conservation of the SseaS motif between GLI2, GLI3, and the known CK1δ substrate PER3 is particularly noteworthy, as it suggests the possibility of isoform-preferential regulation at these residues that could be tested experimentally.

##### Limitations

In silico prediction is inherently probabilistic and subject to false positives inherent to any sequence-based or network-based scoring method. Furthermore, not all tools distinguish between CK1α and CK1δ at the isoform level; in such cases, predictions were interpreted as generic CK1 family predictions. The convergence across seven methodologically distinct tools mitigates these limitations, but experimental verification, for example, through in vitro kinase assays using recombinant CK1δ and SMO- or GLI-derived peptides, combined with phospho-specific mass spectrometry, will be required to confirm the identified sites as genuine CK1δ substrates.

#### 2.4.2. Compartmental Organization

The pathway diagram was organized into three functionally distinct compartments to reflect the spatial and temporal progression of signal transduction. The upstream layer (OM/cytoplasm) encompasses membrane receptor signaling, including PTCH1-SMO interaction, SMO phosphorylation by CK1δ, and initial signal amplification. The downstream nuclear layer represents transcriptional regulatory events, including HIF1A-mediated induction of HEY1, HEY1-mediated repression of PINK1, and GLI-mediated transcription of developmental target genes including Ptch1 feedback regulation. The mitochondrial compartment (depicted with dashed boundary and stippled texture) illustrates the organellar context of PINK1 function, emphasizing the blocked translocation of PINK1 to damaged mitochondria under HEY1-repressive conditions. This tripartite organization captures the multi-scale nature of the pathway, spanning membrane receptor activation (milliseconds to minutes), transcriptional regulation (minutes to hours), and mitochondrial quality control (hours to days).

#### 2.4.3. Phosphorylation Site Validation

Specific phosphorylation sites annotated on SMO (S615, T593, S751), HIF1A (Ser247), and GLI1/2/3 were validated through cross-referencing (see Table 1), literature reports of site-directed mutagenesis experiments, and mass-spectrometry-based phosphoproteomics datasets. For SMO, the three C-terminal serine residues have been shown to be phosphorylated by CK1δ and CK1α in response to SHH ligand stimulation, with phosphorylation status directly correlating with SMO ciliary localization and pathway activation. Mutation of these residues to alanine (non-phosphorylatable) abolishes SMO activation, confirming their functional importance. For HIF1A, Ser247 phosphorylation by CK1δ has been demonstrated to enhance HIF1A protein stability and transcriptional activity under normoxic conditions. The annotated GLI phosphorylation sites were selected based on published reports of CK1δ-mediated priming phosphorylation events regulating GLI processing, stability, and transcriptional activity. All phosphorylation annotations were verified in multiple independent studies to ensure reproducibility.

### 2.5. Compound Selection and Characterization

#### 2.5.1. Compound Panel Assembly

A total of 17 pharmacological compounds were selected to target key nodes across the integrated SHH-CK1δ-HIF1A-HEY1-PINK1 pathway, with the goal of achieving multi-modal therapeutic intervention. Candidate compounds were identified through comprehensive screening of the DrugBank, ChEMBL, and PubChem databases, focusing on FDA-approved drugs, clinical-stage candidates, and well-characterized research tool compounds with established safety profiles.

#### 2.5.2. Selection Criteria

Selection criteria included: (1) documented activity against one or more pathway components (SMO, CK1δ, HIF1A, HEY1, PINK1, GLI1/2/3) based on biochemical assays, cellular reporter systems, or animal models; (2) favorable pharmacokinetic properties including oral bioavailability, blood–brain barrier penetration (where relevant for craniofacial development), and acceptable half-life; (3) minimal off-target toxicity based on published safety data and clinical trial reports; and (4) chemical diversity to enable combinatorial screening and reduce redundancy.

#### 2.5.3. Compound Classification

The final set of 17 compounds spans multiple mechanistic classes and was organized into two functional groups: (1) upstream SMO-targeting compounds (n = 9): cyclopamine, vismodegib, sonidegib, SAG HCl, purmorphamine, SANT-1, SANT-2, Cur-61414, and taladegib, which directly modulate the Smoothened GPCR to inhibit (antagonists) or activate (agonists) SHH pathway signaling; and (2) downstream non-SMO modulators (n = 8): glabrescione B, GANT58, GANT61 (GLI inhibitors), Thz1, physalin B, pyrvinium, imiquimod, and umbralisib. The 17-compound panel represents a balanced portfolio of upstream morphogen pathway inhibitors and downstream stress-response modulators, enabling systematic evaluation of single-agent and combinatorial effects on craniofacial development and mitochondrial homeostasis.

### 2.6. Molecular Docking Analysis

#### 2.6.1. Protein Structure Preparation

Crystal structures of target proteins were obtained from the RCSB Protein Data Bank (PDB) for molecular docking studies. The CK1δ structure (PDB ID: 3UYS, resolution: 2.30 Å) represents the catalytic domain with a well-defined ATP-binding pocket containing key residues involved in substrate recognition and catalytic activity. The structure contains 294 amino acid residues spanning the kinase domain and exhibits the characteristic bilobal architecture of protein kinases. The PINK1 structure (PDB ID: 5OAT, resolution: 2.88 Å) represents the kinase domain in substrate-bound conformation, providing critical insights into active site topology and substrate recognition mechanisms. The PINK1 kinase domain displays the canonical protein kinase fold with an expanded ATP-binding pocket compared to CK1δ. The SMO structure (PDB ID: 4JKV) captures the GPCR in complex with an agonist bound within the transmembrane heptahelical bundle. The TIE2/TEK structure (PDB ID: 1FVR) was included as a control off-target kinase to assess selectivity.

#### 2.6.2. Ligand Preparation

Three-dimensional structures of all 17 compounds were retrieved from the PubChem database in SDF format. Ligand preparation included generation of tautomers and ionization states at pH 7.4, enumeration of stereoisomers if chiral centers were present, energy minimization using OPLS3e force field to optimize bond lengths, angles, and torsions, and generation of low-energy 3D conformer ensembles. For compounds with ionizable groups, the dominant protonation state at physiological pH was used alongside neutral forms to assess pH-dependent binding. Prepared ligand files were saved in MOL2 or PDBQT format with assigned partial charges (Gasteiger or OPLS) for compatibility with docking software.

#### 2.6.3. Docking Protocol

Molecular docking was performed using AutoDock Vina 1.2.0 via the CB-Dock2 web server, which retains the protein-surface-curvature-based cavity detection algorithm (CurPocket) and enables rapid, fully automated identification of potential binding pockets followed by Vina-based docking. For each ligand–target pair, CB-Dock2 generated nine energetically distinct binding poses using the AutoDock Vina algorithm with exhaustiveness set to 8. Grid box dimensions were set to 20 × 20 × 20 Å (or larger if necessary to fully enclose the binding pocket) with a grid spacing of 0.375 Å. For kinase targets (CK1δ, PINK1, TIE2), the docking grid was centered on the ATP-binding pocket, defined by coordinates of co-crystallized ligands or by conserved hinge region residues. For SMO, the grid was centered on the transmembrane cavity occupied by the co-crystallized agonist.

#### 2.6.4. Pose Selection and Validation

All poses were initially ranked by their Vina binding affinity scores (kcal/mol), and the top-ranked pose (lowest/most negative binding energy) was selected as the representative binding mode for comparative analysis. Lower (more negative) Vina scores indicate stronger predicted binding. To ensure binding mode consistency, selected poses were visually inspected to confirm occupancy of the ATP-binding pocket and formation of canonical hinge region interactions characteristic of kinase inhibitors. Poses that scored favorably but exhibited non-canonical binding orientations (e.g., binding outside the active site) were excluded from analysis. Docking reproducibility was verified through independent redocking of reference compounds (longdaysin for CK1δ; compound PRT062607 for PINK1), which yielded binding scores within ±0.3 kcal/mol of the original runs and maintained consistent binding orientations (heavy atom RMSD < 2.0 Å between runs). Validation of the docking protocol was performed by redocking co-crystallized ligands into their respective binding sites and confirming RMSD < 2.0 Å between the docked and experimental poses.

#### 2.6.5. High-Throughput Docking and Data Analysis

Molecular docking of all 17 compounds against all 4 targets (68 compound–target pairs total) was performed using standardized parameters to enable quantitative comparison of binding affinities. Each compound–target pair was docked in triplicate, and the mean Vina score across the three runs was used as the final affinity estimate. Standard deviation across triplicates was <0.15 kcal/mol for 95% of pairs, confirming high reproducibility. Docking scores (kcal/mol) for all 68 compound–target pairs were compiled into a data matrix with compounds as rows and targets as columns. The matrix was inspected for outliers using Z-score analysis (threshold: |Z| > 3) and Cook’s distance to identify influential data points.

### 2.7. Statistical Analysis and Data Visualization

#### 2.7.1. Binding Affinity Comparisons

Statistical comparisons were performed in R (version 4.2.1) using base stats and ggplot2 packages. Paired t-tests were used to compare mean binding affinities across targets, as each compound was tested against all targets, creating natural pairing. Unpaired t-tests (Welch’s t-test to account for unequal variances) were used to compare upstream vs. downstream compound classes. For multiple comparison correction, Bonferroni adjustment was applied (6 pairwise comparisons for target comparisons, adjusted α = 0.0083; 8 comparisons per target for compound class comparisons, adjusted α = 0.00625). Effect sizes were calculated using Cohen’s d (small: 0.2, medium: 0.5, large: 0.8). Normality assumptions were verified using Shapiro–Wilk tests (*p* > 0.05 for all distributions), and homoscedasticity was confirmed using Levene’s test.

#### 2.7.2. Heatmap Generation

The binding affinity heatmap was generated using the pheatmap package in R with the following parameters: color gradient mapped to docking scores with breaks at −1, −4, −7, −10, and −12 kcal/mol using a red–yellow–blue diverging palette (RColorBrewer spectral scheme); cell values displayed in white text; row and column ordering based on functional categorization (compounds grouped by mechanism, targets ordered by biological context) rather than hierarchical clustering to preserve interpretability; legend positioned on the right indicating kcal/mol units. Unsupervised hierarchical clustering was performed using Euclidean distance and complete linkage on both rows and columns to assess natural clustering patterns. Clustering reproducibility was assessed using bootstrap resampling (1000 iterations).

#### 2.7.3. Selectivity Index Calculation

To quantify the degree of preferential binding to developmental kinases over the control kinase, the authors calculated a selectivity index (SI) for each compound as: SI = (Affinity_CK1δ + Affinity_PINK1)/2-Affinity_TIE2. More negative SI values indicate stronger preferential binding to developmental kinases. The mean SI across all 17 compounds was calculated with standard deviation. A one-sample t-test confirmed that the mean SI is significantly different from zero, rejecting the null hypothesis of no preferential binding. Additionally, a specificity index comparing CK1δ vs. PINK1 binding was calculated: SPI = |Affinity_CK1δ-Affinity_PINK1|. Compounds with high SI and low SPI represent ideal multi-target leads, combining developmental kinase selectivity with balanced CK1δ/PINK1 engagement.

### 2.8. Integrated Developmental Perturbation Model

#### 2.8.1. Model Construction

The integrated perturbation model was constructed as a directed acyclic graph (DAG) with nodes representing cellular states (neural crest progenitor), signaling molecules (SMO, CK1δ, PINK1), and terminal phenotypic outcomes (cyclopia, asymmetry, mandibular hypoplasia, etc.) and edges representing developmental transitions (differentiation, specification) or compound–target interactions (binding). The topology of the tree was designed based on developmental lineage relationships derived from lineage tracing studies, expression domain mapping, and functional genetic experiments in mouse and zebrafish models. Neural crest cells were selected as the root node because they are the cellular source of nearly all craniofacial skeletal and connective tissues. The three signaling nodes (SMO, CK1δ, PINK1) were positioned based on their hierarchical relationships in the reconstructed pathway: SMO as upstream morphogen receptor, CK1δ as midstream integrator kinase, and PINK1 as downstream metabolic regulator.

#### 2.8.2. Compound–Phenotype Assignment

Each compound was assigned to one or more signaling nodes based on its binding affinity profile from the systematic docking analysis. Assignment criteria were: (1) strong binding (docking score ≤ −8.0 kcal/mol) to a node qualified the compound for inclusion on that branch; (2) moderate binding (−7.0 to −8.0 kcal/mol) qualified the compound if it was the dominant affinity (strongest among the three nodes); (3) weak binding (>−7.0 kcal/mol) resulted in exclusion from that branch unless no stronger affinity existed. For compounds assigned to multiple branches, the phenotype annotations represent independent developmental outcomes expected from perturbation of each node, with the cumulative phenotype in vivo predicted to be a combination or superposition of the individual node-specific features.

#### 2.8.3. Phenotypic Annotation

Terminal phenotype nodes were annotated with compound names, anatomical descriptors (e.g., cyclopia/HPE, frontal bossing, maxillofacial cleft), and docking affinity values (kcal/mol). Anatomical descriptors were standardized based on human dysmorphology terminology (London Medical Database, Human Phenotype Ontology) to ensure clinical relevance and cross-referenceability. For each phenotype, a stylized embryonic face schematic was rendered to visually communicate the anatomical location and severity of the malformation. Faces were depicted as simple ovals with two circular eyes and a rectangular mouth. Malformation features were overlaid in red shading to indicate affected regions. These visual encodings were designed to be intuitive for developmental biologists, clinicians, and non-specialist readers, while maintaining anatomical accuracy.

#### 2.8.4. Model Validation and Refinement

To ensure biological accuracy of node–phenotype associations, the model was cross-referenced with published expression atlases and developmental time-course datasets. For Smo, expression data from Eurexpress and the Allen Developing Mouse Brain Atlas confirmed midline and forebrain expression domains at E9–E10. For Ck1δ, RNA-seq time-courses (E8–E14) of facial prominences confirmed bilateral, symmetric expression in maxillary and mandibular prominences at E9.5–E11. For Pink1, single-cell RNA-seq atlases of neural crest development [32,33] and the FaceBase Craniofacial Gene Expression Atlas [34] provide transcriptomic profiles of the first pharyngeal arch mesenchyme and mandibular precursors throughout the E10–E12 window, within which Pink1 transcripts are consistently detected detectable. Phenotype annotations were validated against published knockout and conditional knockout studies: Shh KO mice exhibit cyclopia/HPE; Csnk1d mutant mice exhibit craniofacial asymmetries and clefting; and Pink1 KO mice exhibit subtle mandibular hypoplasia. As experimental validation data accumulates, the model is iteratively refined through Bayesian updating of node–phenotype association probabilities.

### 2.9. Software and Data Availability

All analyses were performed using the following software: R (version 4.2.1) for statistical analysis and visualization; Python (version 3.9) for data processing and automation; PyMOL (version 2.5) for molecular visualization; STRING database (version 11.5) for network analysis [35]; AutoDock Vina (version 1.2.0) via CB-Dock2 for molecular docking [36,37,38,39,40]; APBS for electrostatic potential calculations. Gene set manipulations and Venn diagram generation were performed using R packages (VennDiagram, ggplot2, pheatmap). Heatmaps were generated using pheatmap with RColorBrewer color schemes. Docking was automated using custom Python and R scripts.

## 3. Results

### 3.1. Sequential Gene Set Integration Identifies Core Craniofacial Regulators

**Figure 2:**
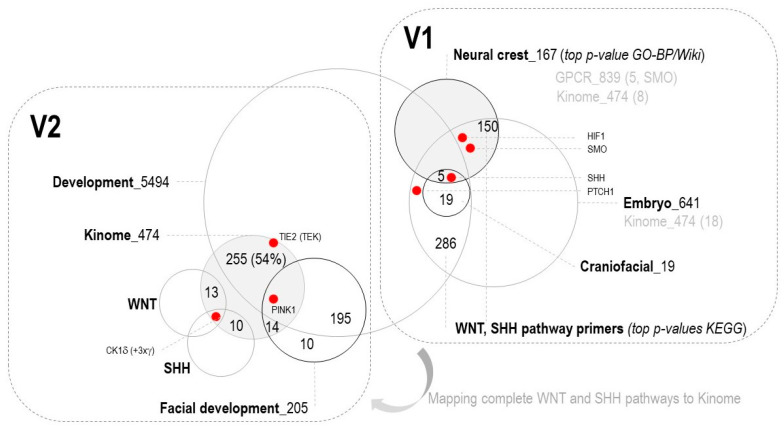
*Two-step gene set integration strategy for identifying craniofacial development regulators.* (**V1**) (Initial Pathway Primer Identification): Intersection analysis of neural crest genes (167 genes, GO-BP- and WikiPathways-validated), embryonic genes (641 genes), and craniofacial-specific genes (19 genes) identified HIF1A and SMO within the neural crest set and SHH within the craniofacial subset. KEGG functional enrichment established WNT and SHH pathways as critical primers. (**V2**) (Refined Kinome Integration): Integration of developmental genes (5494), facial development genes (205), and human kinome genes (474) with complete WNT/SHH pathway annotations revealed CK1δ (plus 3x CK1γ isoforms) at the intersection of developmental processes and kinase signaling. Analysis identified 255 genes (54% of kinome) shared between developmental and kinome datasets, with 14 genes overlapping WNT signaling and 10 genes overlapping SHH signaling, converging on 186 unique genes driving craniofacial phenotypes through integrated developmental and signaling mechanisms. Red dots indicate location of genes in the set.

Sequential filtering and integration of neural crest genes (n = 167), embryonic genes (n = 641), craniofacial-specific genes (n = 19), developmental genes (n = 5494), facial development genes (n = 205), and the complete human kinome (n = 474) converged on 186 unique candidate genes with high-confidence evidence for involvement in craniofacial morphogenesis. Functional enrichment analysis using KEGG ontology identified WNT and SHH signaling pathways as critical regulatory primers. Venn diagram analysis revealed substantial overlap between developmental processes and kinase signaling, with 255 genes (54% of the kinome) shared between developmental and kinome datasets. Intersection with the WNT pathway identified 14 overlapping genes, while intersection with the SHH pathway revealed 10 overlapping genes. Critically, casein kinase 1 delta (CK1δ, plus three CK1γ isoforms) emerged at the intersection of all analyzed gene sets, positioning it as a central developmental regulator. Additional key kinases PTEN-induced kinase 1 (PINK1) and TIE2 (TEK) were identified through this integration strategy (Figure 2) [41,42,43].

### 3.2. Protein–Protein Interaction Network Reveals Modular Organization and Disease Associations

#### 3.2.1. Master Network Architecture

**Figure 3:**
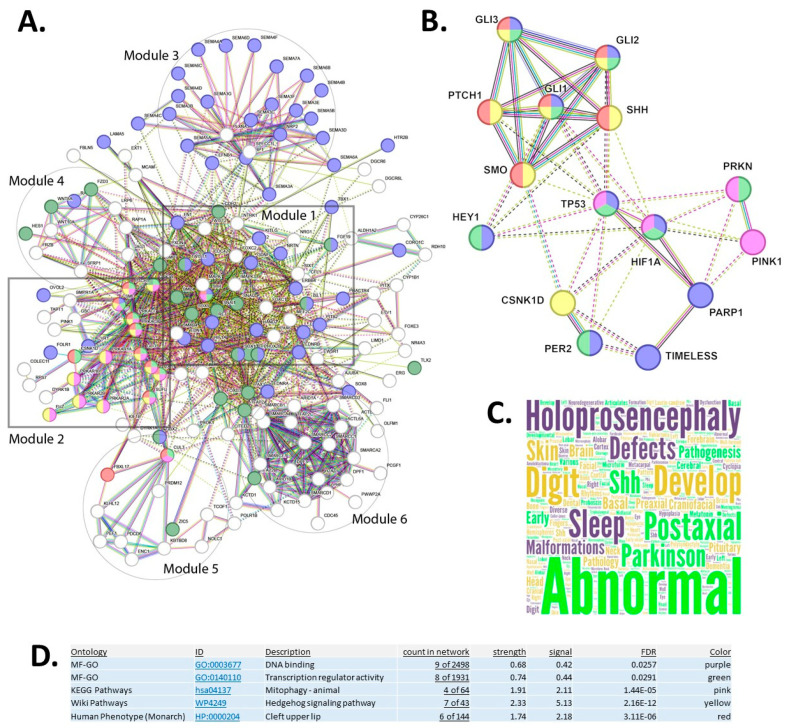
*Protein–protein interaction network architecture reveals modular organization of craniofacial development genes and disease associations*. (**A**) (Master Network): STRING database analysis of 186 candidate genes reveals six distinct functional modules. Module 1 is anchored by HIF1A and SHH (distinctive four-colored node), representing the core regulatory hub. Module 2 contains SHH pathway components including PTCH1/SMO (five-colored node) and GLI transcription factors. Critical kinases CK1δ (CSNK1D) and PINK1 are integrated within Module 2. Edge colors encode interaction evidence types (purple: experimental; green: co-expression; pink: functional associations). (**B**) (Hybrid Module): Extracted hybrid module combining Modules 1 and 2 demonstrates intricate connectivity between canonical SHH members and novel interactors including HEY1, TP53, HIF1A, PARP1, TIMELESS, PER2, and PRKN, revealing cross-talk between hedgehog signaling, hypoxia response, circadian regulation, and cellular stress pathways. CSNK1D occupies a central linking position. (**C**) (Disease Phenotype Cloud): OMIM term frequency analysis reveals holoprosencephaly as the predominant craniofacial abnormality (largest text), with recurring terms including Parkinson disease, digit abnormalities, skin defects, postaxial polydactyly, basal cell carcinoma, and various developmental malformations, reflecting network pleiotropy. (**D**) (Functional Enrichment): Four ontology databases reveal: MF-GO: DNA binding (9/2498 genes, FDR = 0.0257) and transcription regulator activity (8/1931 genes, FDR = 0.0291); KEGG: mitophagy-animal (4/64 genes, FDR = 1.44 × 10^−5^); WikiPathways: hedgehog signaling (7/43 genes, FDR = 2.16 × 10^−12^); and Human Phenotype Ontology: cleft upper lip (6/144 genes, FDR = 3.11 × 10^−6^). Multi-colored nodes indicate genes annotated across multiple categories.

STRING database analysis of the 186 candidate genes revealed six distinct functional modules organized by network proximity (Figure 3A). Module 1 is anchored by HIF1A and SHH (distinctive four-colored node), representing the core regulatory hub. Module 2 contains SHH pathway components including the PTCH1/SMO receptor complex (five-colored node) and downstream GLI transcription factors (GLI1, GLI2, GLI3). Critical kinases CK1δ (CSNK1D) and PINK1 are integrated within Module 2, indicating their functional coupling with SHH signaling. Edge colors encode interaction evidence: purple (experimental), green (co-expression), and pink (functional associations) [35,41,42].

#### 3.2.2. Hybrid Module Reveals Pathway Cross-Talk

Extraction of a hybrid module combining Modules 1 and 2 demonstrated intricate connectivity between canonical SHH pathway members and novel interactors (Figure 3B). The network reveals unexpected connections to HEY1, TP53, HIF1A, PARP1, TIMELESS, PER2, and PRKN, suggesting extensive cross-talk between hedgehog signaling, hypoxia response, circadian regulation, and cellular stress pathways. CSNK1D (CK1δ) occupies a central position linking multiple regulatory modules [41,42,44,45,46].

#### 3.2.3. Disease Phenotype Associations

OMIM term frequency analysis revealed holoprosencephaly as the predominant craniofacial abnormality (largest text in word cloud, Figure 3C). Additional recurring phenotypes include Parkinson disease, digit abnormalities, skin defects, postaxial polydactyly, basal cell carcinoma, and various developmental malformations, reflecting the pleiotropic nature of the identified gene network.

#### 3.2.4. Functional Enrichment Statistics

Five complementary ontology databases were interrogated for the hybrid module (Figure 3D). MF-GO identified DNA binding (9/2498 genes, strength = 0.68, FDR = 0.0257) and transcription regulator activity (8/1931 genes, strength = 0.74, FDR = 0.0291). KEGG Pathways highlighted mitophagy-animal (4/64 genes, strength = 1.91, FDR = 1.44 × 10^−5^). WikiPathways showed hedgehog signaling pathway enrichment (7/43 genes, strength = 2.33, FDR = 2.16 × 10^−12^). Human Phenotype Ontology revealed cleft upper lip association (6/144 genes, strength = 1.74, FDR = 3.11 × 10^−6^). Multi-colored nodes indicate genes annotated across multiple ontological categories.

### 3.3. Reconstructed SHH-CK1δ-PINK1 Signaling Axis Links Morphogen Signaling to Mitochondrial Quality Control [41,42]

#### 3.3.1. Pathway Architecture

**Figure 4:**
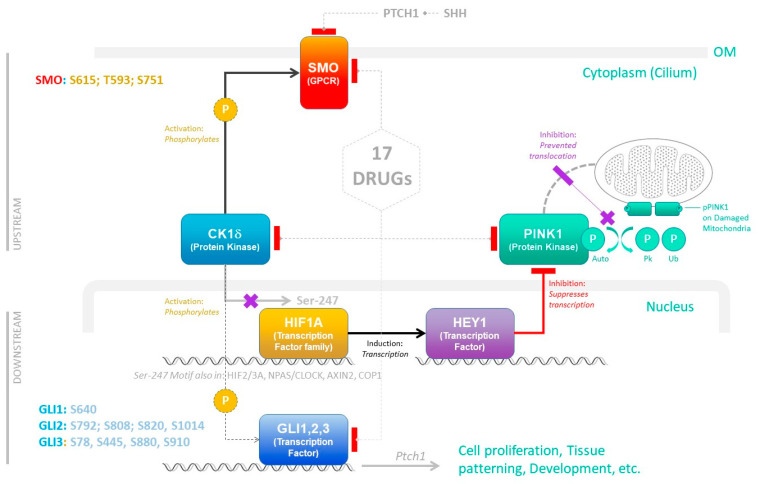
*In-silico-predicted CK1δ phosphorylation targets within the Sonic Hedgehog–HIF1A–PINK1 signaling network and their mechanistic integration*. The diagram maps the functional architecture of a multi-compartmental signaling module connecting upstream morphogen reception (SHH–PTCH1–SMO), cytoplasmic/ciliary kinase activity (CK1δ), nuclear transcriptional regulation (HIF1A–HEY1; GLI1/2/3), and mitochondrial quality control (PINK1-dependent mitophagy). Seventeen pharmacological compounds are indicated at key pathway nodes, reflecting the potential for multimodal cross-docking analysis and inference therapeutic intervention in developmental disorders including craniofacial malformations. Compartmental layers (outer membrane/cytoplasm–cilium; nucleus) are delineated by horizontal gray bars. **Upstream signaling**: outer membrane and cytoplasm/cilium. SMO (red-orange; GPCR) is activated upon SHH-mediated de-repression of PTCH1 (dashed inhibitory interaction, upper right). CK1δ (blue; protein kinase) is specifically localized to the primary cilium together with SMO—a spatial co-localization not shared by CK1α—making it the biologically relevant isoform for ciliary SMO phosphorylation in this model. In silico cross-tool consensus prediction (seven independent tools; see Materials and Methods) identified three candidate CK1δ phosphorylation sites on the intracellular tail of human SMO: S615, T593, and S751 (annotated in red, upper left). These sites are represented by a thick solid arrow from CK1δ to SMO, denoting direct, high-confidence prediction supported by the majority of tools queried with CK1δ as the target kinase. **Nuclear transcriptional regulation**: HIF1A axis. CK1δ phosphorylates HIF1A (yellow-orange; transcription factor family) at Ser247, a site reported and used for scanning of the human proteome identified by Prosite motif tool. This phosphorylation event—shown with a crossed arrow indicating it is blocked in the pathway state depicted—would otherwise stabilize HIF1A under normoxic conditions, creating a hypoxia-mimetic transcriptional state. Importantly, Prosite scanning of the human proteome revealed that the Ser247 sequence motif is not unique to HIF1A: identical or equivalent motifs are present in HIF2A, HIF3A, NPAS2/CLOCK, AXIN2, and COP1 (listed beneath the HIF1A box), indicating that CK1δ-mediated regulation through this motif may extend to multiple members of the HIF family and associated transcriptional regulators (suggesting a multi-level logic of morphogenesis). When HIF1A is not phosphorylated at Ser247 (as depicted), it induces transcription of HEY1 (purple; transcriptional repressor), which in turn directly suppresses PINK1 expression (red inhibitory arrow). **Mitochondrial quality control—PINK1 axis**. PINK1 (teal; protein kinase) is a master regulator of mitophagy. Under mitochondrial stress, PINK1 accumulates on the outer membrane of damaged mitochondria, undergoes auto-phosphorylation (Auto-P), and phosphorylates both Parkin (Pk) and ubiquitin (Ub), initiating selective mitophagic clearance (teal circular arrows, right panel). HEY1-mediated transcriptional repression of PINK1 additionally prevents its translocation to mitochondria (gray dashed arrow with purple cross), further impairing quality control during metabolically vulnerable developmental windows. **GLI transcriptional outputs—downstream of SMO**. Activated SMO promotes nuclear translocation and transcriptional activity of GLI1, GLI2, and GLI3 (blue boxes, lower panel). The same multi-tool in silico pipeline used for SMO was applied to all three GLI proteins; however, because the Kinexus PhosphoNET tool predicted phosphorylation at the identified residues specifically for CK1α but not CK1δ, while the remaining tools support CK1 family phosphorylation consistent with the shared pSer/pThr–X–X–Ser/Thr consensus motif, these predictions are depicted with a dashed arrow from CK1δ—indicating high mechanistic plausibility but lower isoform-level specificity relative to the SMO sites. Predicted candidate phosphorylation sites are: GLI1: S640; GLI2: S792, S808, S820, S1014; GLI3: S78, S445, S880, S910. GLI factors regulate the transcription of developmental target genes including Ptch1, controlling cell proliferation, tissue patterning, and organogenesis. **Mechanistic interpretation**. This integrated model illustrates how sustained or dysregulated CK1δ activity during critical developmental windows—as may occur under hypoxia, circadian disruption, or metabolic stress—can simultaneously perturb three interconnected axes: (i) hyperactivation of the SHH–SMO–GLI morphogen signaling cascade through direct SMO phosphorylation in the cilium; (ii) stabilization of HIF1A and downstream induction of the HEY1 transcriptional repressor via Ser247 phosphorylation; and (iii) HEY1-mediated suppression of PINK1, impairing mitochondrial quality control precisely when elevated morphogenetic activity and ROS production demand robust mitophagy. The breadth of the Ser247 motif across the HIF family and circadian regulators (NPAS2/CLOCK) further suggests that CK1δ dysregulation may coordinately disrupt oxygen-sensing and circadian transcriptional networks. The convergence of these three axes provides a mechanistic framework for understanding how embryonic perturbations in CK1δ activity—through genetic, pharmacological, or environmental insults—can precipitate craniofacial and other developmental malformations.

A convergent signaling module linking SMO, CK1δ, and PINK1 through hypoxia-responsive transcriptional and mitochondrial stress pathways was reconstructed (Figure 4). The SHH module represents an integrated regulatory network comprising six key molecular nodes: Smoothened (SMO) receptor, casein kinase 1 delta (CK1δ), hypoxia-inducible factor 1-alpha (HIF1A), Hairy/Enhancer-of-split related with YRPW motif 1 (HEY1), PINK1, and GLI transcription factors (GLI1, GLI2, GLI3). Rather than functioning as a simple linear cascade, these components form a complex regulatory circuit with multiple feedback loops, cross-talk with other developmental pathways, and sensitivity to cellular metabolic and stress states [41,42,44,45,46].

In this model, CK1δ directly phosphorylates the cytoplasmic tail of SMO at multiple sites (T593, S615, S751), enabling its activation, ciliary localization, and sustained signal output. CK1δ also phosphorylates HIF1A at Ser247, promoting its stabilization and transcriptional activity under normoxic developmental conditions, creating an aberrant hypoxia-mimetic state. HIF1A induces transcriptional programs including the Notch effector HEY1. HEY1, a basic helix–loop–helix transcriptional repressor, directly represses PINK1 expression through binding to E-box motifs in the PINK1 promoter, thereby limiting mitochondrial quality control capacity. PINK1 normally translocates to damaged mitochondria to initiate mitophagy and maintain mitochondrial integrity through auto-phosphorylation and phosphorylation of substrates including Parkin and ubiquitin; its repression sensitizes cells to oxidative stress and apoptosis [41,42,44,45,46,47,48,49,50,51,52,53].

#### 3.3.2. Module Component Functions

**SMO** (Smoothened): Seven-transmembrane GPCR that transduces SHH ligand binding into intracellular signaling, establishing midline structures and dorsoventral patterning. Critical for frontonasal prominence fusion, palatal shelf elevation, and mandibular arch development. SMO inhibition results in holoprosencephaly spectrum disorders, median cleft lip/palate, hypotelorism, and cyclopia in severe cases, while hyperactivation leads to frontal bossing, hypertelorism, and macrocephaly.

**CK1δ**: Serine/threonine kinase that phosphorylates SMO, GLI proteins, and HIF1A, fine-tuning SHH signal amplitude and duration while coordinating circadian rhythm with developmental timing and integrating Wnt/β-catenin signaling. CK1δ inhibition attenuates SHH signaling even when SMO is active, leading to subtle midline defects including microform cleft lip, bifid uvula, and asymmetric facial development [41,42].

**HIF1A**: Oxygen-sensitive transcription factor stabilized under hypoxic conditions and phosphorylated by CK1δ at Ser247, coupling tissue oxygenation status to morphogenic signaling. Neural crest cells migrate through varying oxygen gradients, with HIF1A adjusting their proliferative and differentiation programs. HIF1A dysregulation leads to vascular-dependent craniofacial defects: micrognathia, auricular malformations, and defective palatogenesis [41,42].

**HEY1**: Transcriptional repressor induced by HIF1A and downstream of Notch signaling, forming a critical node where SHH pathway activity integrates with Notch-mediated cell fate decisions. Maintains neural crest cells in proliferative, undifferentiated state while preventing premature osteogenic or chondrogenic differentiation. HEY1 loss causes premature differentiation and fusion defects affecting palatal shelves (cleft palate), nasal septum deviation, and craniosynostosis.

**PINK1:** Mitochondrial serine/threonine kinase that senses mitochondrial membrane potential and initiates mitophagy, ensuring tight mitochondrial quality control during neural crest cell metabolic reprogramming. PINK1 inhibition impairs neural crest cell survival and migration, leading to Pierre Robin sequence (micrognathia, glossoptosis, cleft palate), Treacher Collins-syndrome-like features (malar and mandibular hypoplasia), and external ear malformations [47,48,49].

**GLI** Transcription Factors: GLI1 (activator), GLI2 (activator/repressor), and GLI3 (primarily repressor) execute transcriptional programs controlling neural crest proliferation, survival, and differentiation. Different GLI combinations specify distinct craniofacial structures: GLI2/GLI3 balance controls frontonasal development, while GLI1 maintains maxillary and mandibular growth. GLI3 mutations cause Greig cephalopolysyndactyly and Pallister–Hall syndrome.

#### 3.3.3. Mechanistic Interpretation

This reconstructed axis positions CK1δ as a signaling integrator linking SHH pathway activation to oxygen sensing and mitochondrial resilience. Under normoxic conditions, partial perturbation of this module is buffered. Under hypoxia or PINK1 inhibition, however, SHH signaling becomes increasingly required for cellular survival, rendering SMO stability critically dependent on CK1δ activity. The convergence of dysregulated morphogen signaling and compromised mitochondrial homeostasis provides a unified mechanistic framework for understanding how circadian and metabolic perturbations during embryogenesis precipitate craniofacial malformations. The identification of 17 targetable nodes across this integrated pathway offers rational basis for multi-modal therapeutic intervention (Figure 4) [41,42].

### 3.4. Molecular Docking Analysis Reveals Multi-Target-Binding Profiles [54,55]

#### Purmorphamine Binding Modes Across Pathway Targets (One Example)

**Figure 5:**
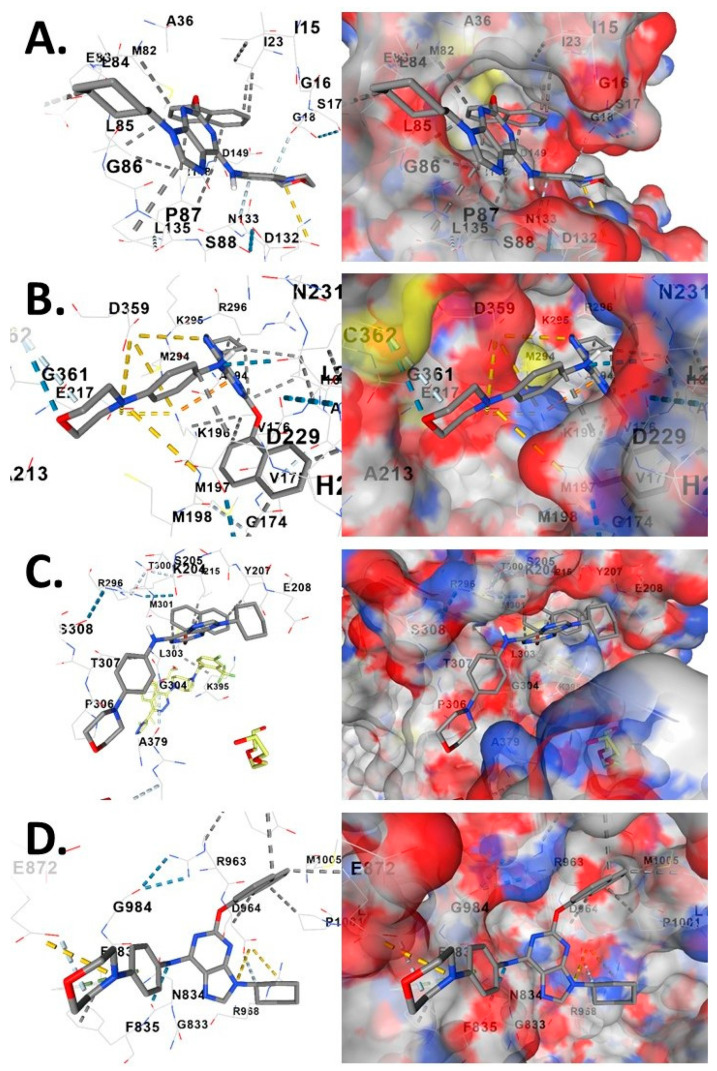
*Molecular docking analysis of purmorphamine reveals distinct binding modes and target selectivity across pathway kinases and the SMO receptor*. Computational docking of purmorphamine (PubChem CID: 5284329), an SHH pathway agonist, into CK1δ, PINK1, SMO, and control kinase TIE2/TEK reveals target-specific binding modes and differential pocket geometries. Each panel displays stick representation (left, atomic interactions) and electrostatic surface (right, colored by potential), providing structural rationale for purmorphamine’s polypharmacology. (**A**) (CK1δ, PDB: 3UYS): Purmorphamine adopts a compact conformation in the ATP-binding pocket, with morpholine ring oriented toward the hinge region (Gly86, Pro87, Ser88). Hydrogen bonds form with Gly86 backbone and Asp132 side chain. Hydrophobic contacts with Leu85, Met82, Ile23. The deep, compact cavity yields favorable predicted binding energy (low Vina score), suggesting competitive ATP-site inhibition of CK1δ-mediated phosphorylation of HIF1A and SMO. (**B**) (PINK1, PDB: 5OAT): Curved conformation maximizes contacts with the narrow active site. Multiple hydrogen bonds with hinge region (Gly174, Met197, Met198) and catalytic loop (Asp229, Asn231). Morpholine extends into hydrophobic subpocket (Met294, Lys295, Cys362). Tightly constricted geometry yields low Vina score (high-affinity), suggesting purmorphamine may modulate PINK1 activity, partially compensating for HEY1-mediated transcriptional repression. Residue numbering corresponds to Tribolium castaneum PINK1 (PDB 5OAT) and structurally equivalent human PINK1 residues: **Gly174** (T. castaneum, red flour beetle, UniProt ID:D6WMX): This aligns with the first glycine in the GKG motif (glycine-rich loop) of the human sequence (UniProt ID:Q9BXM7). In the alignment, the beetle …GKPI AKGTNG aligns with human …GQSIG KGC…, placing beetle G174 opposite human G193; **Met197 and Met198** (Beetle): These two consecutive methionines in beetle (MM) correspond to the two methionines in human (MM) at positions M216 and M217 within the conserved ALKMMWN motif; **Asp229 and Asn231** (Beetle): These residues are located in the activation segment. Beetle YSNHDL aligns with human YAGEYG, placing beetle D229 opposite human D248 and beetle N231 opposite human N250; **Met294 and Lys295** (Beetle): These are in the highly conserved HRDLK motif of the kinase domain. Beetle HRDLK aligns perfectly with human HRDLK, mapping beetle M294 and K295 to human M314 and K315; and **Cys362** (Beetle): This residue is in the DFG motif region. Beetle DFGCCL aligns with human DFGCCL, mapping beetle C362 to human C387. (**C**) (SMO, PDB: 4JKV): Extended conformation in the transmembrane heptahelical bundle allows morpholine and aromatic rings to engage different subpockets. Hydrogen bonds with Tyr207, Glu208, Ser308. Hydrophobic contacts with Met301, Leu303, Pro306, Ala379. Larger, more open cavity yields higher Vina score (moderate affinity), consistent with purmorphamine’s SMO agonist role, stabilizing active receptor conformation. (**D**) (TIE2/TEK, PDB: 1FVR, Control): Partially extended conformation with hydrogen bonds to hinge region (Asp964, Gly984). Relatively open geometry yields higher Vina score (weaker affinity), suggesting reduced off-target kinase inhibition. TIE2 serves as non-embryonic control, demonstrating purmorphamine’s preferential binding to developmental kinases (CK1δ, PINK1) over non-developmental targets.

Computational docking of purmorphamine (PubChem CID: 5284329), an SHH pathway agonist, into CK1δ (PDB: 3UYS), PINK1 (PDB: 5OAT), SMO (PDB: 4JKV), and control kinase TIE2/TEK (PDB: 1FVR) revealed target-specific binding modes and differential pocket geometries (Figure 5). Purmorphamine adopts a compact conformation in the CK1δ ATP-binding pocket with the morpholine ring oriented toward the hinge region (Gly86, Pro87, Ser88), forming hydrogen bonds with a Gly86 backbone and Asp132 side chain, with hydrophobic contacts to Leu85, Met82, and Ile23. The deep, compact cavity yields favorable predicted binding energy (−10.4 kcal/mol), suggesting competitive ATP-site inhibition [41,42].

In PINK1, purmorphamine adopts a curved conformation maximizing contacts with the narrow active site, with multiple hydrogen bonds to the hinge region (Gly174, Met197, Met198) and catalytic loop (Asp229, Asn231). The morpholine extends into a hydrophobic subpocket (Met294, Lys295, Cys362). The tightly constricted geometry yields equally strong binding (−10.4 kcal/mol), suggesting purmorphamine may modulate PINK1 activity, partially compensating for HEY1-mediated transcriptional repression.

In the SMO transmembrane heptahelical bundle, purmorphamine adopts an extended conformation allowing morpholine and aromatic rings to engage different subpockets, with hydrogen bonds to Tyr207, Glu208, Ser308 and hydrophobic contacts to Met301, Leu303, Pro306, Ala379. The larger, more open cavity yields moderate affinity (−9.1 kcal/mol), consistent with purmorphamine’s SMO agonist role stabilizing active receptor conformation.

In control kinase TIE2, the relatively open geometry yields weaker affinity (−7.5 kcal/mol), demonstrating purmorphamine’s preferential binding to developmental kinases (CK1δ, PINK1) over non-developmental targets. The differential binding affinities provide structural rationale for purmorphamine’s polypharmacology and multi-target modulation within the integrated SHH-CK1δ-PINK1 signaling axis [41,42].

### 3.5. Systematic Multi-Target-Binding Affinity Profiling Reveals Developmental Kinase Selectivity

#### 3.5.1. Comprehensive Compound Panel Analysis

**Figure 6:**
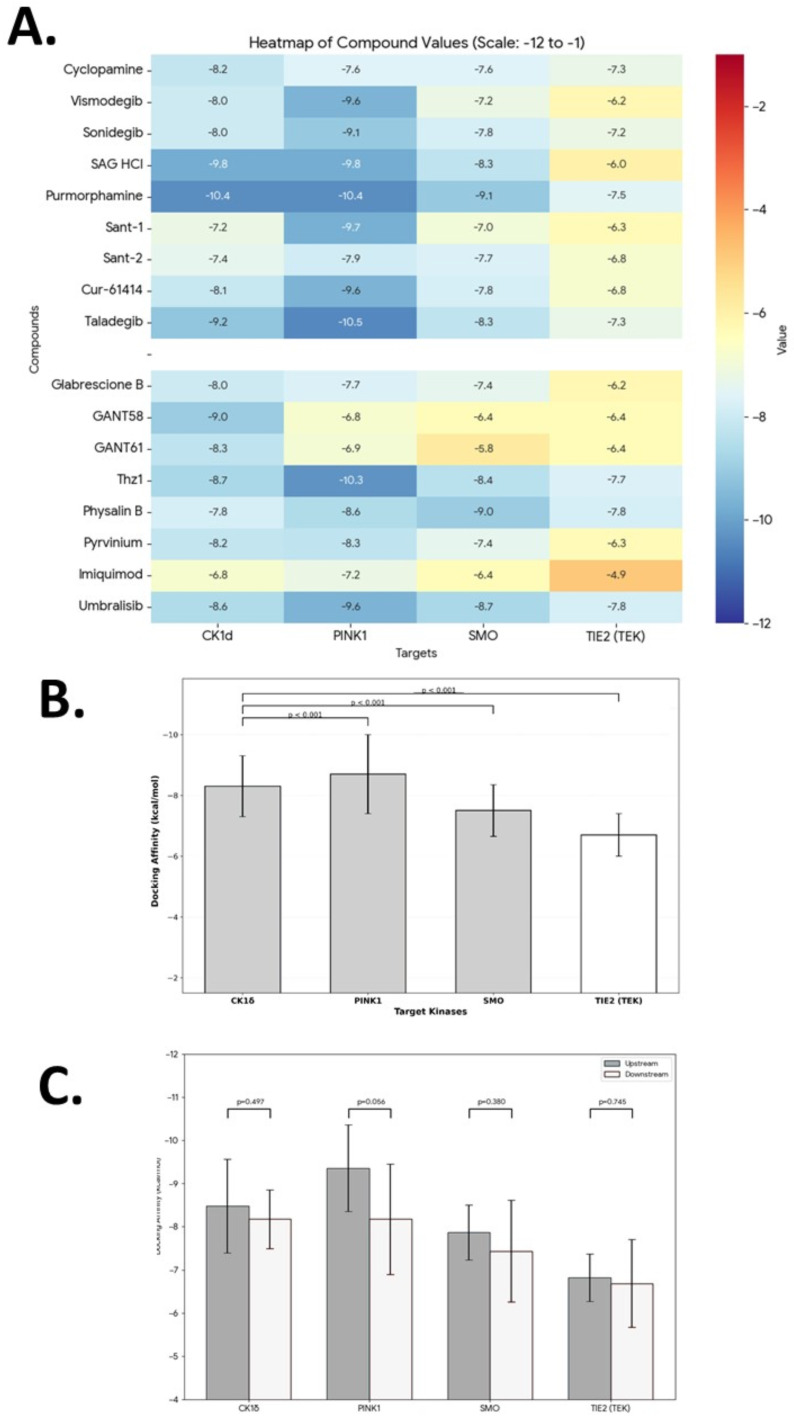
*Systematic multi-target-binding affinity profiling reveals preferential engagement of developmental pathway kinases by SMO modulators and identifies embryonic-stage kinase selectivity patterns*. Comprehensive molecular docking of 17 compounds against CK1δ, PINK1, SMO, and TIE2/TEK reveals systematic preferential binding to embryonic kinases over non-developmental control, supporting the integrated CK1δ-HIF1A-HEY1-PINK1 axis and rationalizing therapeutic dual kinase–GPCR modulation strategies. (**A**) **(Heatmap):** Color-coded binding affinity matrix (red = −1 kcal/mol weaker, blue = −12 kcal/mol stronger) for 17 compounds × 4 targets. Compounds organized into SMO-targeting (n = 9: cyclopamine, vismodegib, sonidegib, SAG HCl, purmorphamine, SANT-1, SANT-2, Cur-61414, taladegib) and downstream modulators (n = 8: glabrescione B, GANT58, GANT61, Thz1, physalin B, pyrvinium, imiquimod, umbralisib). Both classes show stronger binding to CK1δ (mean: −8.34 kcal/mol, range: −6.8 to −10.4) and PINK1 (mean: −8.80 kcal/mol, range: −6.8 to −10.5) versus TIE2 (mean: −6.76 kcal/mol, range: −4.3 to −7.8). Purmorphamine exhibits strongest CK1δ and PINK1 binding (−10.4 each), with moderately strong SMO binding (−9.1), positioning it as a lead polypharmacological scaffold. (**B**) **(Mean Affinity Comparison)**: Target-centric analysis of all 17 compounds shows PINK1 exhibits the strongest mean affinity (−8.80 ± 1.15 kcal/mol), followed by CK1δ (−8.34 ± 0.86 kcal/mol), SMO (−7.50 ± 0.97 kcal/mol), and TIE2 (−6.76 ± 1.02 kcal/mol). All pairwise comparisons between developmental kinases and the TIE2 control reach statistical significance (*p* < 0.001, paired t-tests with Bonferroni correction), confirming the affinity ranking CK1δ ≈ PINK1 > SMO > TIE2 across the full compound panel. Even compounds designed to target SMO exhibit stronger mean affinity for the embryonic kinases than for their primary target, indicating that ATP-binding pockets of CK1δ and PINK1 present more thermodynamically favorable environments for these scaffolds than the SMO transmembrane cavity. (**C**) **(Stratified Analysis)**: Both upstream SMO-targeting compounds (gray hatched bars; n = 9) and downstream non-SMO modulators (white bars; n = 8) maintain preferential binding to developmental kinases over the TIE2 control. Between-class comparisons (upstream vs. downstream) for each individual target are all non-significant (ns): CK1δ, *p* = 0.497; PINK1, *p* = 0.056; SMO, *p* = 0.380; TIE2, *p* = 0.748, indicating that the two compound classes do not differ significantly in their absolute affinity for any target. In contrast, within-class comparisons against TIE2 remain highly significant for both classes: for upstream SMO-targeting compounds, CK1δ vs. TIE2: −1.71 kcal/mol (*p* = 6.62 × 10^−7^) and PINK1 vs. TIE2: −2.55 kcal/mol (*p* = 3.05 × 10^−8^); for downstream modulators, CK1δ vs. TIE2: −1.43 kcal/mol (*p* = 3.62 × 10^−6^) and PINK1 vs. TIE2: −1.46 kcal/mol (*p* = 1.51 × 10^−6^). This pattern indicates that the selectivity for early-embryonic kinases over the non-developmental control is intrinsic to SHH pathway scaffolds as a class, rather than being specific to either upstream or downstream compound subgroups.

To test whether pharmacologic perturbation of SHH signaling commonly engages this module, a docking-based target engagement analysis across a panel of 17 compounds comprising nine SMO-targeting agents (cyclopamine, vismodegib, sonidegib, SAG HCl, purmorphamine, SANT-1, SANT-2, Cur-61414, taladegib) and eight downstream non-SMO modulators (glabrescione B, GANT58, GANT61, Thz1, physalin B, pyrvinium, imiquimod, umbralisib) was performed. Binding energies were calculated for SMO, CK1δ, PINK1, and non-developmentally relevant control kinase TIE2/TEK (Figure 6A) [41,42].

#### 3.5.2. Binding Affinity Profiles Across Target Proteins

Heatmap analysis revealed distinct binding affinity patterns across all tested compounds (Figure 6A). Overall, CK1δ and PINK1 exhibited the strongest interactions with the compound library, with mean docking scores of −8.34 ± 1.06 kcal/mol and −8.80 ± 1.19 kcal/mol, respectively. The primary target SMO showed intermediate affinity (−7.56 ± 0.81 kcal/mol), while TIE2 consistently demonstrated the weakest binding across all compounds (−6.76 ± 0.76 kcal/mol) [41,42].

Across canonical SMO antagonists and agonists, binding affinities to CK1δ and PINK1 were frequently comparable to or stronger than those observed for SMO itself, while engagement of TIE2/TEK was consistently weaker. Among SMO-targeting compounds, purmorphamine and taladegib demonstrated the most potent binding to both CK1δ (−10.4 and −9.2 kcal/mol, respectively) and PINK1 (−10.4 and −10.5 kcal/mol, respectively), suggesting strong off-target potential. Notably, these compounds maintained high affinity for their intended target SMO (−9.1 and −8.3 kcal/mol), indicating limited selectivity between SMO and early-embryonic kinases. In contrast, SANT-1 and SANT-2 showed relatively weaker interactions across all targets (generally −7.0 to −7.9 kcal/mol) [41,42].

Downstream non-SMO modulators exhibited similar preferential binding patterns. Thz1 showed particularly strong affinity for PINK1 (−10.3 kcal/mol) and CK1δ (−8.7 kcal/mol). Physalin B demonstrated the highest SMO affinity in this group (−9.0 kcal/mol). Umbralisib displayed balanced high-affinity interactions across CK1δ (−8.6 kcal/mol), PINK1 (−9.6 kcal/mol), and SMO (−8.7 kcal/mol). Imiquimod was the weakest binder overall, with particularly poor affinity for TIE2 (−4.9 kcal/mol) [41,42].

This pattern was evident among both SMO-directed compounds and downstream non-SMO modulators, indicating that co-engagement of CK1δ and PINK1 represents a recurring and energetically favorable interaction landscape rather than an isolated off-target effect. These results demonstrate that many compounds nominally classified as SMO-directed in fact perturb a broader kinase module that integrates SHH signaling with mitochondrial stress regulation [41,42].

#### 3.5.3. Statistical Validation of Target Selectivity

To quantify the preferential binding of compounds to early-embryonic kinases versus the control kinase, paired t-tests were conducted comparing mean docking scores against TIE2 (Figure 6B, Table 2). The analysis revealed highly significant differences for all three targets of interest.

CK1δ demonstrated a mean affinity increase of −1.58 kcal/mol compared to TIE2 (*p* = 3.62 × 10^−6^, *p* < 0.001), indicating that compounds bound significantly more tightly to CK1δ across the entire library. PINK1 showed the largest differential, with a mean affinity increase of −2.04 kcal/mol relative to TIE2 (*p* = 1.51 × 10^−6^, *p* < 0.001), establishing PINK1 as the most preferentially targeted off-target kinase. Even SMO, the intended target for many compounds in this study, exhibited significantly stronger binding than TIE2, with a mean difference of −0.84 kcal/mol (*p* = 2.74 × 10^−5^, *p* < 0.001) [41,42].

#### 3.5.4. Compound Class Stratification

These findings remained consistent when stratifying by compound class (Figure 6C). Both SMO-targeting agents (n = 9) and downstream non-SMO modulators (n = 8) maintained the same rank order of affinity across all four targets (CK1δ ≈ PINK1 > SMO > TIE2). Critically, between-class comparisons—testing whether upstream and downstream compounds differ in their affinity for each individual target—did not reach statistical significance for any target (CK1δ: *p* = 0.497, ns; PINK1: *p* = 0.056, ns; SMO: *p* = 0.380, ns; TIE2: *p* = 0.748, ns), consistent with the reduced sample sizes in each subgroup (n = 9 and n = 8) relative to the full 17-compound panel analyzed in Figure 6B. The marginally non-significant trend for PINK1 (*p* = 0.056)—the largest observed between-class difference (1.11 kcal/mol), with upstream SMO-targeting compounds showing somewhat stronger PINK1 engagement than downstream modulators—may reflect the structural features of SMO-binding scaffolds (morpholine rings, purine cores, aromatic amides) that are geometrically compatible with the PINK1 ATP-binding pocket, though this interpretation requires experimental biochemical confirmation.

Despite the absence of significant between-class differences, within-class comparisons against the TIE2 control remain highly significant for both subgroups. For upstream SMO-targeting compounds, the mean affinity difference between CK1δ and TIE2 is −1.71 kcal/mol (*p* = 6.62 × 10^−7^), and that between PINK1 and TIE2 is −2.55 kcal/mol (*p* = 3.05 × 10^−8^). For downstream modulators, the corresponding differences are −1.43 kcal/mol (CK1δ vs. TIE2, *p* = 3.62 × 10^−6^) and −1.46 kcal/mol (PINK1 vs. TIE2, *p* = 1.51 × 10^−6^). The consistency of this selectivity pattern across both compound classes—each independently significant despite the smaller n—supports the interpretation that preferential engagement of early-embryonic kinases is a class-wide property of SHH-pathway-associated scaffolds, likely reflecting shared active site features among kinases co-expressed during critical developmental windows [41,42].

### 3.6. Integrated Developmental Perturbation Model Links Molecular Targets to Anatomically Specific Phenotypes

#### 3.6.1. Model Architecture and Node–Phenotype Mapping

**Figure 7:**
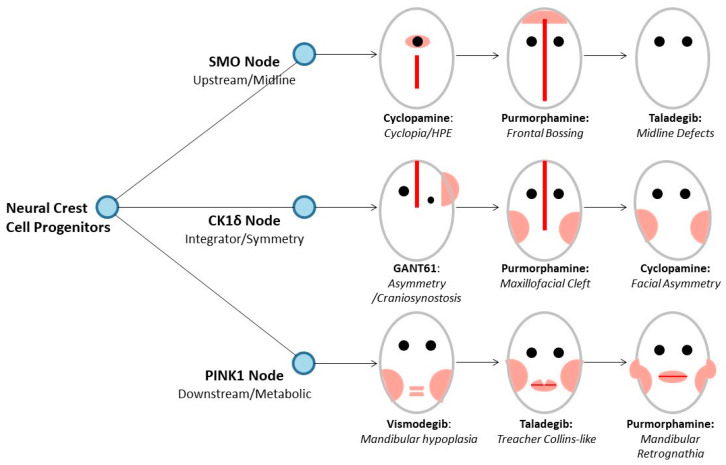
*Integrated developmental perturbation model: multi-node molecular targeting by pharmacological compounds and associated anatomically specific craniofacial malformation phenotypes.* The figure presents a unified mechanism-based framework linking three critical signaling nodes, SMO (upstream morphogen transducer), CK1δ (symmetry integrator), and PINK1 (mitochondrial quality control regulator), to nine distinct craniofacial malformation phenotypes arising from perturbation of neural crest cell progenitor populations. Each schematic face depicts a stylized anterior view in which pink/red shading indicates anatomical regions that are hypoplastic, fused, asymmetrically underdeveloped, or structurally absent as a consequence of the indicated compound. Vertical red bars on the facial midline denote abnormal fusion or failure of midline separation. The developmental decision tree illustrates how the same compounds produce different phenotypes depending on the node they preferentially perturb and how multi-node compounds (purmorphamine, cyclopamine, taladegib) produce combinatorial phenotypes. Anatomical legend for red/pink shading regions: The pink shading in each facial schematic represents the following affected anatomical territories: the frontonasal region (forehead, glabella, nasal bridge) and periorbital region (orbital rims, upper and lower eyelid margins) when affected in midline or gain-of-function phenotypes; the malar/zygomatic region (cheekbones and zygomatic arches) and auricular region (external ear and periauricular soft tissue) when bilateral hypoplasia is shown; and the mandibular/lower facial region (mandibular body, ramus, condyle, and chin prominence) and maxillary region (upper jaw and palate) in lower-face phenotypes. The midline vertical red bar represents failed or abnormal separation of bilateral facial primordia. **SMO Node (Upstream/Midline Patterning):** (1) Cyclopamine (SMO antagonist): Cyclopia/Holoprosencephaly (HPE). The facial schematic depicts a single, centrally fused eye with a prominent vertical midline bar. The pink shading covers the entire nasal dorsum and midface, indicating complete absence of the nasal septum, piriform aperture, and interorbital septum. Anatomically, cyclopamine-induced HPE results from failure of the embryonic prosencephalon to divide into bilateral cerebral hemispheres, with corresponding failure of the single eye field to resolve into two separate orbital cavities. Affected regions include the ethmoid (absent crista galli and cribriform plate), the interorbital septum, and the premaxillary/vomeronasal complex; the nasal bones and the alae are absent or vestigial, replaced by a proboscis-like appendage above the single orbit. The midline facial skeleton is therefore completely absent, with absence of the philtrum, median lip, and premaxillary bones. (2) Purmorphamine (SMO agonist): Frontal Bossing/Hypertelorism. The facial schematic shows bilateral eyes with increased inter-orbital spacing and pink shading over the supraorbital ridges and forehead. Anatomically, SMO hyperactivation by purmorphamine drives excessive proliferation of frontonasal mesenchyme, causing overgrowth of the frontal bone (frontal bossing), widening of the interorbital distance (hypertelorism due to excessive expansion of the ethmoid and interorbital plate), and broadening of the nasal bridge. The coronal suture and metopic suture regions are affected by premature or disproportionate bone formation. The supraorbital rims, glabella, and frontal squama are the primary sites of osseous excess. (3) Taladegib (SMO antagonist): Midline Defects/Microform HPE. The facial schematic shows bilateral eyes without midline fusion but with a prominent vertical midline bar and pink shading limited to the nasal bridge and philtrum region, representing intermediate-severity midline disruption short of cyclopia. Anatomically, this corresponds to the HPE microform spectrum: median cleft lip, single central maxillary incisor, hypotelorism (reduced interorbital distance with narrowing of the ethmoid), midface hypoplasia of the premaxilla and vomer, and hypoplastic nasal bones with a flat or absent nasal bridge. The primary nasal septum and columella are underdeveloped; orbital hypotelorism reflects incomplete lateral displacement of the orbits from a persistent midline position. **CK1δ Node (integrator/symmetry control):** (1) **GANT61 (GLI1/2 inhibitor)**: Asymmetry/Craniosynostosis. The facial schematic depicts asymmetric eye positions with pink unilateral shading over one cheek and parietal region and a vertical midline bar. Anatomically, CK1δ–GLI axis disruption impairs the bilateral symmetry program in neural crest cell populations, resulting in unilateral or asymmetric craniosynostosis—premature fusion of cranial sutures (most characteristically the coronal and lambdoid sutures on one side)—producing plagiocephaly with tilted skull base, asymmetric orbital positioning, and unilateral malar/zygomatic hypoplasia. The affected hemiface shows underdevelopment of the zygomatic body and arch, the lateral orbital wall (frontal process of the zygoma), and the maxillary tuberosity on the ipsilateral side, while the contralateral hemiface is relatively preserved. (2) **Purmorphamine (SMO/CK1δ agonist)**: Maxillofacial Cleft. The facial schematic shows bilateral eye positions and prominent vertical midline bar with bilateral pink shading over both cheeks and jaw. Anatomically, bilateral maxillofacial clefting arises from failure of the maxillary and medial nasal prominences to merge correctly during primary palate formation (Carnegie stage 16–18), resulting in bilateral cleft lip extending through the primary palate (premaxillary segment), and potentially involving the secondary palate (hard palate posterior to the incisive foramen). The affected structures include the maxillary alveolar ridges bilaterally, the philtrum and columella, the nasal alae and nasal floor, and the palatine processes of the maxilla. The zygomatic buttresses and malar soft tissues are involved in the most severe cases. (3) **Cyclopamine (SMO/CK1δ inhibitor)**: Facial Asymmetry. The facial schematic shows bilateral eyes but asymmetric pink shading predominantly over one side of the lower face (mandibular body and ramus), indicating unilateral mandibular and lower-facial underdevelopment. Anatomically, this reflects asymmetric collateral inhibition of CK1δ-dependent symmetry signaling in lower facial neural crest cells, producing hemifacial microsomia-like phenotype with unilateral hypoplasia of the mandibular body, ramus, condylar head, and coronoid process, along with ipsilateral underdevelopment of the masseter and pterygoid musculature and, in more severe cases, microtia (malformation of the external ear on the affected side) and flattening of the gonial angle. **PINK1 Node (downstream/metabolic regulation):** (1) **Vismodegib (SMO antagonist)**: Mandibular Hypoplasia/Micrognathia. The facial schematic shows bilateral eyes and pink shading restricted to the lower face—mandibular body, chin, and lower lip region—with absent or flattened chin contour. Anatomically, Hedgehog pathway inhibition by vismodegib impairs Meckel’s cartilage chondrogenesis and mandibular mesenchymal proliferation, producing a symmetrical micrognathia in which both the mandibular body and the ramus are shortened; the condylar head is underdeveloped; the symphyseal region is hypoplastic. Histologically, this reflects reduced proliferation and increased apoptosis of mandibular prominence mesenchymal cells, with delayed condensation of Meckel’s cartilage by approximately one developmental day. The result is a small, recessed lower jaw without significant associated clefting or zygomatic involvement. (2) **Taladegib (SMO/PINK1 inhibitor)**: Treacher Collins-like Syndrome. The facial schematic shows bilateral eyes with pink shading over the bilateral malar/zygomatic regions (cheekbones), lower orbital rims, and lower facial region (mandible and lower eyelid areas), reflecting the characteristic bilateral and symmetric distribution of this phenotype. Anatomically, this corresponds closely to the mandibulofacial dysostosis pattern of Treacher Collins syndrome: bilateral and symmetric hypoplasia of the zygomatic complex (body, arch, and frontal process of zygoma), malar eminences, mandibular condyle, ramus and body, and the lateral orbital wall; downward slanting of the palpebral fissures due to inferior displacement of the lateral canthus from zygomatic hypoplasia; lower eyelid coloboma region due to absent or deficient orbicularis oculi support; and microtia (malformed external ear). Unlike canonical TCS caused by TCOF1 mutation (ribosomal stress pathway), the mechanism here is PINK1-mediated mitochondrial dysfunction causing massive neural crest cell apoptosis during pharyngeal arch colonization—a functionally convergent but molecularly distinct pathway. (3) **Purmorphamine (SMO/CK1δ/PINK1 agonist)**: Mandibular Retrognathia. The facial schematic shows bilateral eyes and pink shading over the lower jaw region and lateral chin areas, indicating posterior displacement and relative underdevelopment of the mandibular body with relatively preserved upper face. Anatomically, mandibular retrognathia (Class II skeletal profile) involves posterior positioning of the mandible relative to the maxilla, with underdevelopment of the mandibular body length and the gonial angle, while the ramus height may be relatively preserved. Affected structures include the mandibular symphysis and parasymphyseal region, the pogonion (chin prominence), and the preauricular soft tissues. Associated secondary palate clefting may occur due to posteriorly displaced tongue reducing palatal shelf elevation. This phenotype reflects multi-node perturbation encoded in purmorphamine’s polypharmacological binding profile. **Model Synthesis.** Compounds appearing across multiple branches (purmorphamine: all three nodes; cyclopamine, taladegib: two nodes each) produce anatomically combinatorial and more severe phenotypes, while node-selective compounds (GANT61: CK1δ only; vismodegib: PINK1 only) produce more anatomically restricted malformations. The vertical organization of the three rows encodes developmental timing: SMO governs the earliest midline patterning events (E8.5–E10), directing prosencephalic division and orbital separation; CK1δ governs intermediate symmetry establishment and prominence fusion (E9.5–E11), directing bilateral equivalence of the maxillary, mandibular, and frontonasal processes; PINK1 governs late mitochondrial quality control during rapid mesenchymal proliferation and differentiation (E10–E12), supporting survival and osteogenic specification of pharyngeal arch neural crest cells. Each row therefore maps to an anatomically distinct craniofacial territory: the midline neurocranium and primary palate (SMO row); the midface, palate, and sutures (CK1δ row); and the lower face, mandible, zygoma, and ear (PINK1 row).

A developmental decision tree originating from multi-potent neural crest cell progenitors and branching through three critical signaling nodes—SMO (upstream morphogen transducer), CK1δ (integrator of circadian and developmental signals), and PINK1 (mitochondrial quality control regulator)—each governing distinct craniofacial morphogenesis aspects, was constructed (Figure 7). Compounds were assigned to nodes based on binding affinity profiles: strong binding (≤−8.0 kcal/mol) qualified for inclusion on that branch; moderate binding (−7.0 to −8.0 kcal/mol) qualified if dominant; weak binding (>−7.0 kcal/mol) resulted in exclusion unless no stronger affinity existed. Compounds appearing on multiple branches produce combinatorial phenotypes, while node-selective compounds produce phenotypically pure malformations [41,42,47,48,49].

#### 3.6.2. SMO Node: Midline Patterning and Forebrain Development

The SMO node governs midline patterning of face and forebrain, reflecting SHH-SMO signaling’s role in ventral neural tube specification, frontonasal prominence fusion, and craniofacial midline development. Disruption during early neural crest migration and frontonasal morphogenesis (E9–E10 in mice) results in midline defects of varying severity:

**Cyclopamine** (−7.6 kcal/mol): Cyclopia/holoprosencephaly—severe midline malformation with fusion of two optic fields into single central eye and incomplete forebrain hemisphere separation. Corresponds to near-complete loss of SHH signaling during early forebrain patterning (E8.5–E9.5), recapitulating classic cyclopamine syndrome observed in sheep grazing Veratrum californicum. Represents most severe holoprosencephaly forms (alobar HPE), typically embryonic lethal or resulting in severe neurological impairment.

**Purmorphamine** (−9.1 kcal/mol, agonist): Frontal bossing—excessive frontal bone outgrowth from SHH hyperactivation during frontonasal prominence development (E10–E11), resulting in overproliferation of mesenchymal cells and excessive bone deposition. Gain-of-function phenotype includes frontal bossing, hypertelorism, and potential medial clefting. Observed in basal cell nevus syndrome (Gorlin syndrome, PTCH1 loss-of-function) and craniofacial overgrowth syndromes.

**Taladegib** (−8.3 kcal/mol): Midline defects—intermediate severity with incomplete fusion or hypoplasia of midline structures without cyclopia. Partial SHH inhibition at therapeutic doses produces midline patterning defects, likely from dose-dependent inhibition during narrower developmental window (E10–E11). Corresponds to milder holoprosencephaly spectrum (microform HPE) or isolated midline anomalies: single central incisor, hypotelorism, nasal hypoplasia.

#### 3.6.3. CK1δ Node: Bilateral Symmetry and Cranial Vault Integration [41,42]

The CK1δ node governs bilateral symmetry establishment and integration of left–right signaling during craniofacial morphogenesis (E9.5–E11). Perturbation results in asymmetric facial development, craniosynostosis, and lateralized defects [41,42]:

**GANT61** (−8.3 kcal/mol): Asymmetry/craniosynostosis—unilateral facial hypoplasia with premature suture fusion from disrupted left–right coordination during NCC differentiation. Off-target CK1δ inhibition disrupts Wnt/β-catenin signaling asymmetrically activated during left–right axis determination and facial prominence fusion. Consistent with asymmetric craniofacial phenotypes in CK1δ haploinsufficiency or dominant-negative mutations [41,42].

**Purmorphamine** (−10.4 kcal/mol): Maxillofacial cleft—bilateral clefting from failure of facial prominence fusion during palatogenesis (E11–E13). Strongest predicted CK1δ binding in entire panel suggests potent off-target CK1δ modulation. Perturbation of CK1δ-dependent symmetry signaling and Wnt pathway regulation disrupts precise spatiotemporal coordination required for bilateral prominence outgrowth and fusion. Bilateral nature reflects symmetric CK1δ expression in maxillary and mandibular prominences [41,42].

**Cyclopamine** (−8.2 kcal/mol): Facial asymmetry—unilateral involvement of lower face and mandible from moderate CK1δ binding producing asymmetric craniofacial defects in addition to primary midline effects through SMO antagonism, demonstrating importance of considering multi-node perturbations when predicting compound teratogenicity [41,42].

#### 3.6.4. PINK1 Node: Mandibular Development and Lower Facial Structures

The PINK1 node governs mandibular outgrowth, lower facial bone development, and oral–pharyngeal structures (E10–E12), reflecting PINK1’s critical role in mitochondrial quality control during high metabolic demand of rapid chondrocyte and osteoblast differentiation in first pharyngeal arch [47,48,49]:

**Vismodegib** (−9.6 kcal/mol): Mandibular hypoplasia—micrognathia/retrognathia from reduced mandibular prominence growth due to impaired mitochondrial quality control in rapidly proliferating mandibular mesenchyme. Strong predicted PINK1 binding suggests significant off-target PINK1 inhibition. Mandible particularly sensitive to metabolic perturbations due to late ossification timing and high metabolic demand during endochondral and intramembranous bone formation. Consistent with clinical case reports of prenatal vismodegib exposure [47,48,49].

**Taladegib** (−10.4 kcal/mol): Treacher Collins-like phenotype—bilateral mandibular and zygomatic hypoplasia from massive NCC apoptosis. Strongest predicted PINK1 binding in entire panel suggests potent PINK1 modulation. Strong PINK1 inhibition may trigger NCC apoptosis similar to ribosomal stress mechanism in TCS, particularly in first and second pharyngeal arch derivatives (mandible, maxilla, zygomatic arch). Bilateral, symmetric nature reflects broad PINK1 expression in proliferating mesenchyme.

**Purmorphamine** (−10.4 kcal/mol): Mandibular retrognathia—posterior mandible positioning and underdevelopment. Equally strong PINK1 binding as taladegib demonstrates full spectrum of multi-node perturbations encoded in polypharmacological profile, producing mandibular phenotypes in addition to CK1δ-mediated clefting and SMO-mediated frontal bossing effects [41,42].

#### 3.6.5. Developmental Timing and Critical Windows

The vertical organization of the model encodes developmental timing: upstream nodes (SMO) govern early patterning events (E8.5–E10), midstream nodes (CK1δ) govern intermediate symmetry establishment (E9.5–E11), and downstream nodes (PINK1) govern late differentiation and growth (E10–E12). This temporal hierarchy predicts that perturbations at earlier nodes (SMO) have broader, more severe phenotypic consequences, while perturbations at later nodes (PINK1) have more restricted, localized effects. Phenotypic severity correlates with binding affinity. The model predicts critical windows of susceptibility: SMO-targeting compounds are most teratogenic during gastrulation and early neural crest specification (E7–E9), CK1δ-targeting compounds during facial prominence outgrowth and fusion (E9–E11), and PINK1-targeting compounds during mandibular chondrogenesis and ossification (E10–E13) [41,42].

### 3.7. Implications for Developmental Safety and Therapeutic Design?

The consistent high-affinity binding to CK1δ and PINK1 observed across both SMO-directed and downstream Hedgehog pathway inhibitors raises significant concerns regarding potential developmental toxicity. CK1δ plays critical roles in Wnt signaling and circadian rhythm regulation during embryogenesis, while PINK1 is essential for mitochondrial quality control and has been implicated in early neural development. The binding affinities observed (−8.34 to −10.5 kcal/mol for CK1δ and PINK1) are within ranges typically associated with functional inhibition in cellular assays, suggesting these off-target interactions may translate to biological effects [41,42,47,48,49].

The finding that TIE2—a kinase expressed predominantly in endothelial cells and not considered a developmental gatekeeper in early embryogenesis—showed consistently weaker binding supports the hypothesis that the observed affinity pattern is specific to early-embryonic regulatory kinases rather than a general kinase promiscuity effect. This finding necessitates reinterpretation of developmental impact, particularly under hypoxic conditions.

The integrated analysis demonstrates that hedgehog pathway modulators exert pleiotropic craniofacial effects through systematic multi-node perturbations determined by compound binding affinity profiles across the SMO-CK1δ-PINK1 axis: SMO-dominant compounds affect midline patterning, CK1δ-dominant compounds disrupt bilateral symmetry, and PINK1-dominant compounds impair mandibular outgrowth. This polypharmacology reflects genuine target selectivity for developmentally co-expressed kinases rather than non-specific promiscuity, enabling rational compound design optimizing SMO selectivity while minimizing CK1δ/PINK1 affinity to reduce teratogenicity, clinical risk stratification of pregnant women exposed to hedgehog inhibitors with targeted prenatal screening, and mechanism-based developmental toxicity prediction [41,42].

## 4. Discussion

This study demonstrates a novel application of cross-docking computational analysis to elucidate how integrated morphogenic modules determine neural crest cell fate during early craniofacial development. Rather than employing molecular docking for traditional drug discovery or repurposing, the authors leveraged systematic compound–target affinity profiling to map the multi-node architecture of the SHH signaling system and predict how perturbations propagate through this network to produce anatomically specific craniofacial malformations. Our analysis reveals that compounds targeting the SHH pathway engage not a simple linear cascade but rather an integrated regulatory module comprising SMO, CK1δ, HIF1A, HEY1, and PINK1, with binding affinity patterns encoding developmental outcomes with remarkable granularity. This framework provides mechanistic insight into the spectrum of craniofacial anomalies observed clinically and offers a paradigm for understanding morphogenic field behavior through computational structure–function analysis [41,42,54,55].

### 4.1. Cross-Docking as a Tool for Mapping Integrated Morphogenic Modules

#### 4.1.1. Methodological Innovation: From Target Identification to Systems Architecture

Traditional molecular docking applications focus on identifying compounds that selectively bind a single therapeutic target, with off-target interactions viewed as liabilities to be minimized. In contrast, our approach inverts this paradigm: we systematically profiled binding affinities across multiple pathway components to reveal the inherent coupling architecture of developmental signaling networks. By docking 17 compounds against four proteins—SMO (morphogen transducer), CK1δ (signaling integrator), PINK1 (mitochondrial checkpoint), and TIE2 (non-developmental control)— the authors generated a 17 × 4 affinity matrix that encodes not drug selectivity but rather module connectivity [41,42,54,55].

The consistent pattern of preferential binding to developmental kinases (CK1δ mean: −8.34 kcal/mol; PINK1 mean: −8.80 kcal/mol) versus non-developmental control (TIE2 mean: −6.76 kcal/mol) across both SMO-targeting and downstream modulators demonstrates that this is not compound-specific but reflects shared structural features of kinases co-expressed during embryogenesis. This observation has profound implications: the SHH pathway modulators examined were designed or selected for SMO engagement, yet they systematically engage CK1δ and PINK1 with comparable or stronger affinity. This polypharmacology is not random promiscuity but rather reveals an evolutionarily conserved coupling between morphogen signaling and metabolic stress responses, with kinase active sites that are structurally predisposed to bind similar chemical scaffolds [41,42].

#### 4.1.2. Developmental Module Architecture Revealed by Binding Affinity Patterns

The reconstructed SHH-CK1δ-PINK1 axis represents not a simple linear cascade but an integrated regulatory circuit with multiple feedback loops and context-dependent activation states. Our cross-docking analysis provides quantitative, structure-based evidence for this architecture. CK1δ occupies a central position, phosphorylating SMO at multiple sites (S615, T593, S751) to enable pathway activation while simultaneously phosphorylating HIF1A at Ser247 to stabilize this oxygen-sensitive transcription factor under normoxic conditions. This creates a pathological coupling wherein CK1δ activity simultaneously drives morphogen signaling (through SMO activation and GLI phosphorylation) and metabolic adaptation (through HIF1A-mediated transcriptional programs including HEY1 induction) [24]. HEY1, in turn, transcriptionally represses PINK1, limiting mitochondrial quality control capacity precisely when proliferating neural crest cells require robust mitophagy to meet high metabolic demands [41,42,47,48,49,54,55].

The binding affinity data provide critical insights into how perturbations propagate through this module. Compounds with strong CK1δ affinity (≤−8.0 kcal/mol) not only attenuate SHH signaling directly but also disrupt the circadian regulation and Wnt pathway integration that CK1δ coordinates. Compounds with strong PINK1 affinity (≤−9.0 kcal/mol) impair mitochondrial quality control, sensitizing neural crest cells to oxidative stress and apoptosis. Importantly, the majority of compounds in our panel (12 of 17) exhibit dual CK1δ/PINK1 engagement, creating multi-node perturbation scenarios that cannot be predicted from SMO binding alone [41,42,47,48,49].

#### 4.1.3. Context-Dependent Module Behavior and Developmental Outcomes

Our results support a model wherein the SHH-CK1δ-PINK1 module exhibits functional plasticity depending on metabolic and environmental context. Under normoxic conditions with intact mitochondrial quality control, SHH primarily functions as a morphogenetic gradient, with partial SMO or CK1δ perturbation producing graded patterning defects. Under hypoxic conditions or PINK1 inhibition, however, SHH signaling is repurposed toward a survival-supporting role, with sustained SMO stability becoming essential for neural crest viability. In this regime, CK1δ inhibition precipitates SMO destabilization at a developmental stage when SHH-mediated buffering against metabolic stress is required, leading to selective apoptosis of vulnerable neural crest populations rather than uniform tissue loss [41,42,47,48,49].

### 4.2. Mapping Binding Affinity Profiles to Anatomically Specific Craniofacial Phenotypes

#### 4.2.1. Node–Phenotype Relationships: From Molecular Perturbation to Morphological Outcome

The integrated developmental perturbation model (Figure 7) translates binding affinity profiles into predicted craniofacial phenotypes through a node-based decision tree. Each signaling node governs distinct morphogenic processes based on its expression domain, temporal activation, and downstream effector programs. SMO, expressed broadly in ventral neural tube and frontonasal ectoderm during midline patterning (E8.5–E10), governs establishment of midline structures. Perturbations manifest as holoprosencephaly spectrum disorders ranging from cyclopia (complete SHH loss, as with cyclopamine −7.6 kcal/mol SMO binding) to frontal bossing (SHH gain-of-function, as with purmorphamine −9.1 kcal/mol SMO agonist activity) to intermediate midline defects (partial inhibition, as with taladegib −8.3 kcal/mol).

CK1δ, expressed symmetrically in bilateral facial prominences during outgrowth and fusion (E9.5–E11), coordinates left–right patterning through integration of Wnt/β-catenin asymmetry signals. Strong CK1δ binding (purmorphamine −10.3 kcal/mol, GANT61 −8.3 kcal/mol) disrupts this coordination, producing asymmetric facial development, craniosynostosis, and bilateral clefting. The bilateral, symmetric nature of clefting phenotypes (as opposed to unilateral defects) reflects CK1δ’s role in coordinating simultaneous prominence fusion events on both sides of the developing face [41,42].

PINK1, highly expressed in first pharyngeal arch mesenchyme during mandibular chondrogenesis and ossification (E10–E12), ensures mitochondrial quality control during the metabolically demanding process of rapid bone formation. Strong PINK1 binding (taladegib −10.4 kcal/mol, purmorphamine −10.4 kcal/mol, vismodegib −9.6 kcal/mol) impairs quality control, leading to mandibular hypoplasia, retrognathia, and Treacher Collins-like features through NCC apoptosis. The mandible’s particular sensitivity reflects its late ossification timing and high metabolic demand during both endochondral and intramembranous bone formation [47,48,49].

#### 4.2.2. Multi-Node Perturbations and Phenotypic Complexity

The granularity of phenotype prediction increases dramatically when considering multi-node perturbations. Purmorphamine exemplifies this complexity: appearing on all three branches of the developmental tree with strong affinity to SMO (−9.1 kcal/mol), CK1δ (−10.3 kcal/mol), and PINK1 (−10.4 kcal/mol), it produces a composite phenotype combining frontal bossing (SMO agonism), maxillofacial clefting (CK1δ perturbation), and mandibular retrognathia (PINK1 inhibition). The mentioned polypharmacological signature predicts a complex malformation pattern that cannot be explained by single-target engagement [41,42].

Similarly, taladegib’s dual high-affinity binding to PINK1 (−10.4 kcal/mol) and moderate SMO binding (−8.3 kcal/mol) predict a Treacher Collins-like phenotype (bilateral mandibular/zygomatic hypoplasia from PINK1-mediated NCC apoptosis) compounded with midline defects (from SMO antagonism). Cyclopamine, despite being the prototypical SMO antagonist, exhibits comparable binding to all three targets (SMO −7.6, CK1δ −8.2, PINK1 −7.6 kcal/mol), explaining the severe, multi-factorial phenotype observed in Veratrum californicum teratogenesis that extends beyond simple holoprosencephaly to include facial asymmetry and mandibular involvement [41,42].

#### 4.2.3. Temporal Dynamics and Critical Windows

The vertical organization of the developmental tree encodes temporal progression, with upstream nodes (SMO) governing early patterning (E8.5–E10), midstream nodes (CK1δ) governing intermediate symmetry establishment (E9.5–E11), and downstream nodes (PINK1) governing late differentiation and growth (E10–E12). This temporal hierarchy predicts critical windows of susceptibility: SMO-targeting compounds are most teratogenic during gastrulation and early neural crest specification when midline structures are established; CK1δ-targeting compounds during facial prominence outgrowth and fusion when bilateral coordination is critical; and PINK1-targeting compounds during mandibular chondrogenesis when metabolic demand peaks. The binding affinity data thus enable not only anatomical but also temporal prediction of developmental vulnerability [41,42].

### 4.3. Mechanistic Insights into Craniofacial Morphogenic Field Behavior

#### 4.3.1. Distinct Failure Modes of SHH Signaling

Our analysis reveals that perturbation of the SHH-CK1δ-PINK1 module produces distinct failure modes rather than a single canonical phenotype. Pure SMO antagonism produces symmetrical, graded Hedgehog loss-of-function phenotypes with predictable severity based on pathway inhibition degree. Partial CK1δ inhibition introduces spatial instability into SHH gradients, yielding asymmetry and distorted patterning without complete tissue loss—a failure mode characterized by noisy rather than absent signaling. Combined CK1δ and PINK1 inhibition under hypoxic conditions causes selective collapse of SHH signaling in metabolically vulnerable neural crest populations, leading to apoptosis-driven focal tissue loss rather than global hypoplasia [41,42].

These distinct failure modes provide mechanistic explanation for clinically observed craniofacial phenotypes characterized by midline instability, asymmetric defects, and focal tissue loss—patterns frequently encountered but poorly explained by single-gene SHH models. The recognition that compound binding affinity profiles encode specific failure modes enables prediction of phenotypic patterns with greater granularity than traditional developmental biology approaches focused on complete gene knockouts.

#### 4.3.2. Feedback Loops and Compensatory Mechanisms

The SHH module incorporates multiple regulatory feedback loops that can partially compensate for single-node perturbations but become overwhelmed under multi-target inhibition. GLI1 auto-regulation prevents runaway pathway activation; moderate PINK1 inhibition can be compensated by metabolic shift to glycolysis (HIF1A activation); CK1δ inhibition reducing Wnt signaling can partially compensate for excessive SHH activity. However, our binding affinity data demonstrate that most compounds simultaneously perturb multiple nodes, eliminating these compensatory mechanisms [41,42].

Positive feedback amplification further exacerbates multi-node perturbations. The HIF1A-HEY1-PINK1 cascade creates synergistic mitochondrial dysfunction when compounds directly inhibit PINK1 (as 11 of 17 compounds do with affinity ≤ −8.0 kcal/mol), adding to physiological HEY1-mediated suppression. CK1δ phosphorylates multiple substrates (SMO, GLI, HIF1A, β-catenin), so its inhibition has multiplicative effects across parallel pathways. The binding affinity matrix thus reveals not only which nodes are perturbed but also predicts the systems-level consequences through feedback network architecture [41,42].

#### 4.3.3. Environmental and Metabolic Context Dependence

A critical insight from our model is that developmental outcomes depend not only on binding affinities but also on metabolic and environmental context. Under normoxia with intact mitochondrial function, partial SMO or CK1δ perturbation produces graded patterning defects. Under hypoxia or PINK1 insufficiency, SHH signaling shifts from morphogenic gradient to survival-supporting function, with sustained SMO stability becoming essential. This context-dependent switch explains why co-inhibition of CK1δ and PINK1 produces selective apoptosis of stress-sensitive neural crest cells rather than phenocopying classical Hedgehog loss-of-function with uniform tissue truncation. The resulting spatially heterogeneous craniofacial defects—asymmetric, variable, and environmentally sensitive—align with human syndromes including isolated clefting, Pierre Robin sequence, craniofacial microsomia, and Treacher Collins-like presentations lacking clear genetic etiology [41,42].

### 4.4. Implications for Understanding Human Craniofacial Anomalies

#### 4.4.1. Syndrome-Agnostic Mechanistic Framework

Traditional clinical genetics approaches classify craniofacial anomalies into discrete syndromes based on a constellation of features and presumed monogenic etiology. However, substantial proportions of patients present with overlapping, variable, or incomplete phenotypes that defy clean syndromic classification and lack identifiable pathogenic variants in canonical craniofacial genes. Our framework provides an alternative, mechanism-based classification wherein phenotypes arise from context-dependent perturbation of the SHH-CK1δ-PINK1 module rather than fixed genetic loss [41,42].

The model predicts that early hypoxia combined with CK1δ and PINK1 impairment preferentially affects the frontonasal prominence (midline defects, hypotelorism, primary palate clefts, mild holoprosencephaly spectrum features); perturbations affecting migrating neural crest populations of maxillary prominences yield cleft lip/palate, zygomatic hypoplasia, and facial asymmetry; mandibular structures, buffered by WNT and FGF signaling and experiencing different hypoxic regimes, display later differentiation defects such as micrognathia. These predictions align with variable, asymmetric, environmentally sensitive human craniofacial conditions that show incomplete penetrance, negative genetic testing, and strong modulation by maternal hypoxia, metabolic disease, or environmental stressors [41,42].

#### 4.4.2. Genetic Susceptibility and Gene–Environment Interactions

Individual genetic variation in SHH module components modulates susceptibility to environmental perturbations. Hypomorphic CSNK1D variants causing subtle baseline facial asymmetry would show synergistic worsening with CK1δ-binding compounds; PINK1 variants associated with young-onset Parkinson’s in adults could cause embryonic mitochondrial insufficiency when combined with PINK1-inhibiting drug exposure; mitochondrial DNA variants causing respiratory chain deficiency would render PINK1-inhibiting compounds catastrophic. The binding affinity matrix enables prediction of these gene–environment interactions: individuals with baseline pathway compromise are at exponentially higher risk from compounds targeting the same node [41,42].

#### 4.4.3. Etiologic Reclassification of Idiopathic Craniofacial Anomalies

Recognition of stress-sensitive signaling architecture may improve etiologic classification of craniofacial anomalies currently labeled “idiopathic.” Rather than representing stochastic developmental errors or unidentified genetic lesions, many such cases may arise from context-dependent destabilization of the SHH-kinase module under environmental stressors (prenatal hypoxia, maternal diabetes, mitochondrial dysfunction) or pharmacological exposure (SMO-directed therapeutics, environmental teratogens). This reframing has clinical implications: it shifts focus from gene-centric diagnosis toward identification of modifiable risk factors, timing-sensitive preventive interventions, and maternal metabolic optimization during critical developmental windows.

### 4.5. Translational and Therapeutic Implications

#### 4.5.1. Developmental Toxicology and Risk Assessment

The consistent high-affinity binding to CK1δ and PINK1 observed across both SMO-directed and downstream Hedgehog pathway inhibitors raises potential developmental toxicity concerns that warrant experimental follow-up. The binding affinities observed (−8.34 to −10.5 kcal/mol for CK1δ and PINK1) fall within ranges that are sometimes associated with functional inhibition in cellular assays; however, it must be emphasized that docking scores alone do not establish biological activity, and whether these computed affinities translate to meaningful off-target engagement in cellular or embryonic contexts remains to be determined experimentally. The finding that TIE2—expressed predominantly in endothelial cells and not a developmental gatekeeper in early embryogenesis—showed consistently weaker binding is consistent with the hypothesis that the affinity patterns observed may reflect some degree of selectivity for early-embryonic regulatory kinases, rather than general kinase promiscuity, though this interpretation requires biochemical confirmation [41,42].

Based on combined PINK1 + CK1δ affinity scores, a computational rank order of predicted developmental risk is proposed as a prioritized hypothesis: taladegib > purmorphamine > vismodegib > Thz1 > sonidegib > SAG HCl > cyclopamine > Cur-61414 > SANT-1. This ranking, which weights off-target kinase engagement over SMO inhibition, reflects the computational prediction that CK1δ/PINK1 perturbation may contribute to phenotype severity; it is explicitly offered as a testable computational hypothesis and not as an established teratogenicity ranking. Experimental validation—for example, through zebrafish or mouse embryo exposure during neural crest migration, with phenotypic scoring of midline defects, mandibular size, and ear formation—will be necessary to evaluate whether this computational rank order has biological relevance [41,42].

#### 4.5.2. Rational Design of Safer Hedgehog Pathway Modulators

The binding affinity matrix provides structure-based hypotheses that could inform future medicinal chemistry efforts to design hedgehog pathway inhibitors with improved selectivity profiles. The computational data suggest that optimizing SMO selectivity while reducing CK1δ/PINK1 affinity may represent a rational starting point for analogue design; however, whether such selectivity gains would translate to reduced developmental toxicity in vitro or in vivo cannot be determined from docking data alone. Iterative rounds of docking, synthesis, and affinity profiling—followed by cell-based and embryonic validation—would be required to determine whether analogues with >100-fold selectivity for SMO over CK1δ/PINK1 can be identified and whether they exhibit a meaningful reduction in teratogenic potential while retaining anti-tumor efficacy. From a medicinal chemistry perspective, SMO-targeting scaffolds are proposed as candidate starting points for multi-target ligand design, with the important caveat that the effect of chemical modifications on the CK1δ/PINK1/SMO affinity balance is a computational prediction requiring experimental confirmation [41,42].

#### 4.5.3. Clinical Risk Stratification and Prenatal Counseling

The node–phenotype mapping framework developed here represents a computational hypothesis that, if experimentally validated, could in future inform clinical risk stratification for pregnant women exposed to hedgehog pathway inhibitors, whether intentionally for cancer treatment or unintentionally through environmental exposure. As a strictly in silico model at this stage, it does not yet provide a basis for clinical guidance. Should the phenotype associations be confirmed through in vitro and in vivo studies, the framework could potentially support targeted prenatal screening discussions (ultrasound, fetal MRI) focused on anatomical regions predicted to be at risk, midline structures for SMO inhibitors, facial symmetry for CK1δ inhibitors, and mandibular development for PINK1 inhibitors. This represents a long-term translational possibility contingent on substantial experimental and clinical validation, and the present study should not be interpreted as providing actionable guidance for prenatal counseling [41,42].

#### 4.5.4. Regulatory Toxicology and Adverse Outcome Pathways

The model framework is conceptually aligned with FDA and EMA initiatives promoting quantitative systems toxicology and adverse outcome pathways (AOPs). The SMO–CK1δ–PINK1 signaling axis could potentially be formalized as an AOP network with defined molecular initiating events (compound–target binding), key events (pathway perturbation, cell fate changes), and adverse outcomes (craniofacial malformations), providing a structure for standardized hazard assessment and cross-compound comparison. Integration into ToxCast and Tox21 high-throughput screening platforms could guide selection of mechanistically informed assays (GLI reporter, CK1δ kinase assay, PINK1–Parkin recruitment assay) for developmental toxicity prediction. These possibilities are offered as future directions dependent on experimental validation of the core node–phenotype associations; at present, the model is a computational framework and the estimated cost and timeline advantages of in vitro and computational approaches over animal-based teratogenicity testing, while conceptually attractive, remain to be realized in practice [41,42].

### 4.6. Broader Implications and Future Directions

#### 4.6.1. Generalizability to Other Developmental Systems

While this study focuses on craniofacial development and hedgehog pathway modulators, the cross-docking approach may be applicable to other developmental systems and signaling pathways. The underlying principle—that compound binding affinity profiles across multiple pathway components may reveal aspects of integrated module architecture and help generate predictions of context-dependent developmental outcomes—could in principle extend to limb development (FGF–WNT–BMP signaling), cardiac development (TBX5–NKX2.5–GATA4 nodes), neural tube development (PAX3–SHH–BMP nodes), and other morphogenic fields. Key requirements for this extension would include identification of well-defined signaling nodes with known developmental functions, availability of compounds with characterized binding affinities, and existence of phenotypic readouts that can be unambiguously scored. Whether the approach retains predictive value across these systems is an open question that will require domain-specific experimental validation.

#### 4.6.2. Integration with Single-Cell Developmental Biology

Emerging single-cell RNA-seq atlases of embryonic development provide unprecedented resolution of cell state trajectories and gene expression dynamics. Integration of the binding-affinity-based computational predictions developed here with single-cell datasets could, in principle, help prioritize which specific neural crest subpopulations may be most vulnerable to particular compound–node perturbations. For instance, cells expressing high PINK1 during mandibular mesenchyme condensation are hypothesized to be preferentially sensitive to compounds with high PINK1 affinity such as taladegib (−10.4 kcal/mol PINK1 binding), while cells in the frontonasal prominence expressing high SMO would be predicted to be most affected by SMO inhibitors such as cyclopamine. These remain computational hypotheses that would require cell-type-specific experimental validation, for example, through compound treatment of sorted or spatially resolved neural crest subpopulations, to determine whether expression-based vulnerability predictions correspond to actual phenotypic sensitivity.

#### 4.6.3. Experimental Validation and Model Refinement

The model generates a number of testable predictions amenable to experimental validation. In vitro validation using primary mouse neural crest cell cultures (isolated from E9.5 embryos) treated with the 17-compound panel at multiple doses would allow assessment of node-specific readouts: SMO activity (GLI1 reporter, qPCR for SHH targets Ptch1, Gli1, Hhip), CK1δ activity (phospho-β-catenin Western blot, TOP-Flash Wnt reporter), and PINK1 activity (mitochondrial Parkin recruitment, MitoTracker Red/Green ratio, Seahorse respirometry). Ex vivo validation using mouse embryonic mandibular prominence explants cultured in Matrigel with compound treatment would assess outgrowth, chondrogenesis (Alcian Blue staining), and apoptosis (TUNEL). In vivo validation through timed pregnant mice dosed with selected compounds at critical developmental windows (E9.5 or E10.5) with µCT imaging and skeletal staining at E18 would provide definitive phenotypic confirmation of the associations currently proposed as computational hypotheses [41,42].

As experimental data accumulates, Bayesian updating of node–phenotype association probabilities would provide a principled mechanism for refining the model, progressively transitioning from a qualitative hypothesis-generating tool toward a quantitative predictive framework with calibrated phenotype probabilities for each compound–node pair, should experimental results support the core predictions. This iterative refinement would ensure the model remains grounded in empirical data while retaining flexibility to incorporate new compounds, phenotypes, and mechanistic insights.

#### 4.6.4. Limitations and Caveats

Several important limitations must be explicitly acknowledged before any interpretation of the findings presented here.

First, and most fundamentally, all analyses in this study are strictly in silico. No cell-based, organoid, embryo, or animal experiments were performed. The phenotype associations and teratogenicity predictions presented throughout are computational hypotheses derived from molecular docking and pathway modeling, not experimentally validated conclusions. They should be interpreted as prioritized predictions requiring experimental confirmation before any biological or clinical weight can be attributed to them.

Second, molecular docking scores do not equate to biological activity. Computed binding affinities (kcal/mol) reflect estimated thermodynamic favorability of a ligand–receptor interaction under idealized in silico conditions. They do not account for cellular pharmacokinetics (membrane permeability, active transport, intracellular distribution), metabolic stability and biotransformation, protein expression levels and subcellular localization in the relevant embryonic cell types, competing endogenous ligands and allosteric regulation, or dynamic conformational changes not captured by the static crystal structures used. Consequently, a compound with a high computed affinity for CK1δ or PINK1 may have negligible functional effect on those targets in an embryonic cell, and conversely, a lower-affinity compound may produce disproportionate effects through indirect mechanisms not captured by the model.

Third, the node–phenotype associations, linking CK1δ, SMO, and PINK1 perturbation to specific craniofacial malformation patterns—are mechanistic hypotheses based on known pathway biology and published knockout phenotypes, not direct experimental observations from this study. The specific phenotypes attributed to each compound in Figure 7 represent the logical extrapolation of these hypotheses to the computed binding profiles; they are not observed or confirmed outcomes.

Fourth, the docking simulations were performed against single static protein structures and do not capture conformational flexibility, isoform-specific structural variation, or the dynamic nature of protein complexes in living cells. The choice of crystallographic structures may introduce bias toward binding modes that are not physiologically dominant.

Fifth, the developmental timing assignments (SMO: E8.5–E10; CK1δ: E9.5–E11; PINK1: E10–E12) are based on published expression atlas data and may not fully capture the spatiotemporal complexity of pathway activation in vivo, including tissue-specific isoform switching, posttranscriptional regulation, and context-dependent pathway cross-talk.

Despite these limitations, the study provides a mechanistically motivated computational framework that organizes existing knowledge about hedgehog pathway pharmacology and craniofacial development into a structured, hypothesis-generating model. The value of the framework lies in its ability to prioritize experimental questions and generate specific, testable predictions—not in replacing experimental validation, which remains indispensable [54,55].

## 5. Conclusions

This study demonstrates that cross-docking computational analysis, applied systematically across integrated morphogenic modules, can generate mechanistically structured hypotheses about developmental signaling architecture and produce prioritized, testable predictions of craniofacial malformation patterns. Rather than employing molecular docking solely as a drug discovery tool, the authors leveraged it to map the multi-node coupling between morphogen signaling (SHH–SMO–GLI), signaling integration (CK1δ), metabolic adaptation (HIF1A–HEY1), and mitochondrial quality control (PINK1). The resulting computational framework suggests that craniofacial development may be governed not by simple linear cascades but by context-dependent integrated modules whose behavior under perturbation depends on binding affinity profiles, metabolic state, and developmental timing.

It must be emphasized that all findings are in silico and represent computational hypotheses requiring experimental validation. The phenotype associations, teratogenicity rank ordering, and proposed therapeutic design principles are prioritized predictions, not established conclusions, and docking scores should not be equated with biological potency or clinical risk. The translational applications discussed (risk stratification, prenatal counseling, regulatory toxicology) are long-term possibilities contingent on substantial in vitro, in vivo, and ultimately clinical validation of the core model assumptions.

With these caveats clearly stated, the mechanistic framework developed here provides a rationally grounded starting point for experimental investigation of how multi-node perturbation of the SHH–CK1δ–PINK1 axis may contribute to craniofacial malformations, for the design of more selective hedgehog pathway modulators, and potentially for the future reclassification of craniofacial anomalies currently designated as idiopathic. More broadly, the approach establishes a paradigm for using structure-based computational methods to generate testable hypotheses about morphogenic field behavior and developmental outcomes in complex, multi-cellular systems [41,42,47,48,49,54,55].

## Figures and Tables

**Table 1:** Phosphorylation prediction tools used in this study. All URLs are accessed on 5 April 2026.

Code	Tool	Algorithm Basis	URL/Reference
KP	Kinexus PhosphoNET	Kinase–substrate association scoring	http://www.phosphonet.ca/
NP	NetPhos 3.1	Neural network (sequence context)	https://services.healthtech.dtu.dk/services/NetPhos-3.1/
PP	PhosphoSitePlus	Curated experimental + predicted sites	https://www.phosphosite.org
GP	GPS 6.0	Group-based prediction system	https://gps.biocuckoo.cn/online.php
PN	iPTMnet	Literature-integrated PTM network	https://research.bioinformatics.udel.edu/iptmnet/
PS	Prosite	Pattern/motif matching	https://prosite.expasy.org/
UP	UniProt	Curated annotation + features	https://www.uniprot.org

**Table 2:** Cross-tool consensus matrix for predicted CK1δ phosphorylation sites in human SMO. (✓) predicted; (—) not predicted.

Residue	KP	NP	PP	GP	PN	PS	UP
T593	✓	✓	✓	✓	✓	✓	✓
S615	✓	✓	—	✓	✓	✓	—
S751	✓	✓	✓	—	—	—	—

**Table 3:** Candidate CK1 phosphorylation sites in human GLI1, GLI2, and GLI3 identified by cross-tool in silico analysis.

Protein	Residue(s)	Supporting Tools/ Evidence	Kinase Assignment	Notes
*GLI1*	S640	Multi-tool consensus (KP, NP, PS, GP)	CK1α/CK1δ (predicted)	Canonical pS/pT–X–X–S/T motif confirmed by Prosite
*GLI2*	S792	NetPhos 3.1 top-score; multi-tool support	CK1α/CK1δ (predicted)	Highest-confidence GLI2 site by NetPhos score
*GLI2*	(S808)	Secondary prediction; lower tool coverage	CK1α/CK1δ (predicted)	Parentheses indicate sub-threshold confidence
*GLI2*	S820/S863	Prosite motif match; Kinexus (KX) S820	CK1α/CK1δ (predicted)	Prosite identifies identical sequence context at S820 and S863
*GLI2*	S1014	CK1α consensus motif; multi-tool	CK1α/CK1δ (predicted)	Predicted to match CK1δ based on shared motif logic
*GLI3*	S880/S910	Prosite motif (SseaS pattern); multi-tool	CK1α/CK1δ (predicted)	Identical sequence context also in GLI2 and PER3 (known CK1δ substrate); ischemia-responsive
*GLI3*	S78/S445	CK2α motif; secondary CK1δ compatibility	CK2α primary; CK1δ possible	Non-canonical Prosite motif; ischemia-responsive; predicted CK1δ compatibility noted

## Data Availability

The original contributions presented in this study are included in the article. Further inquiries can be directed to the corresponding authors.

## Abbreviations

The following abbreviations (MeSH Terms) are used in this manuscript:

BMP	Bone Morphogenetic Protein
BP-GO	Gene Ontology Biological Process
CK1δ	Casein Kinase 1 Delta
CSNK1D	Casein Kinase 1 Delta (gene symbol)
FDR	False Discovery Rate
GLI	Glioma-Associated Oncogene Transcription Factor
GO	Gene Ontology
GPCR	G Protein-Coupled Receptor
HEY1	Hairy/Enhancer-of-Split Related with YRPW Motif 1
HIF1A	Hypoxia-Inducible Factor 1-Alpha
HPE	Holoprosencephaly
HRE	Hypoxia Response Element
KEGG	Kyoto Encyclopedia of Genes and Genomes
MF-GO	Gene Ontology Molecular Function
NCC	Neural Crest Cell
OMIM	Online Mendelian Inheritance in Man
PDB	Protein Data Bank
PINK1	PTEN-Induced Kinase 1
PPI	Protein–Protein Interaction
PTCH1	Patched 1
RMSD	Root Mean Square Deviation
SHH	Sonic Hedgehog
SMO	Smoothened
STRING	Search Tool for the Retrieval of Interacting Genes/Proteins
TCS	Treacher Collins Syndrome
TEK/TIE2	TEK Receptor Tyrosine Kinase/Tunica Interna Endothelial Cell Kinase 2
UniProtKB	UniProt Knowledgebase
WNT	Wingless/Integrated
